# Effectiveness of home-based upper limb rehabilitation in stroke survivors: A systematic review and meta-analysis

**DOI:** 10.3389/fneur.2022.964196

**Published:** 2022-09-09

**Authors:** Sharon Fong Mei Toh, Pei Fen Chia, Kenneth N. K. Fong

**Affiliations:** ^1^Department of Rehabilitation Sciences, The Hong Kong Polytechnic University, Kowloon, Hong Kong SAR, China; ^2^Department of Rehabilitation, Yishun Community Hospital, Singapore, Singapore; ^3^Department of Occupational Therapy, Tan Tock Seng Hospital, Singapore, Singapore

**Keywords:** home-based interventions, hemiparetic upper limb, rehabilitation, stroke, technology

## Abstract

**Background:**

Home-based training is an alternative option to provide intensive rehabilitation without costly supervised therapy. Though several studies support the effectiveness of home-based rehabilitation in improving hemiparetic upper limb function in stroke survivors, a collective evaluation of the evidence remains scarce.

**Objectives:**

This study aims to determine the effects of home-based upper limb rehabilitation for hemiparetic upper limb recovery in stroke survivors.

**Methods:**

The databases of the Cochrane Library, MEDLINE, CINAHL, and Web of Science were systematically searched from January 2000 to September 2020. Only randomized, controlled, and cross-over trials that evaluated the effects of home-based upper limb interventions were selected. The Pedro scale was used to assess the methodological quality of the studies. A meta-analysis of the upper limb function outcomes was performed by calculating the mean difference/standardized mean difference using a fixed/random effect model.

**Results:**

An initial search yielded 1,049 articles. Twenty-six articles were included in the review. The pooled evidence of the meta-analysis showed that home-based upper limb intervention was more effective in improving upper limb function [SMD: 0.28, 95% CI (0.12, 0.44), *I*^2^ = 0%, *p* < 0.001, fixed effect model] than conventional therapy. When comparing two types of home-based interventions, subgroup analysis revealed that home-based technology treatment—electrical stimulation—provided more significant improvement in upper limb function than treatment without the use of technology (SMD: 0.64, 95% CI (0.21, 1.07), *I*^2^ = 0%, *p* = 0.003, random effect model).

**Conclusion:**

The beneficial effects of home-based upper limb interventions were superior to conventional therapy in improving function and perceived use of the hemiparetic upper limb in daily activities. Among the home-based interventions, home-based electrical stimulation seemed to provide the most optimal benefits.

## Introduction

Upper limb disability in stroke survivors poses a significant challenge to rehabilitation practitioners (1). Stroke survivors, carers, and healthcare professionals perceived that further research in upper limb rehabilitation is one of their top priorities (2). Only 15% of the stroke survivors would gain complete functional recovery in their motor functions (3), while 33–60% had little or no function in their hemiplegic arm in the chronic phase (4). Timing, intensity, and task-specific practice are critical elements that facilitate the recovery of the hemiparetic upper limb after a stroke (5–7). However, the intensity of rehabilitation in an outpatient setting after discharge is usually inadequate (8).

Home-based rehabilitation offers an alternative to providing intensive training without costly supervised outpatient rehabilitation (9) and, more importantly, as a buffer during the transition from inpatient to rehabilitation services in the community. Increasingly, technological innovation has been deployed to provide home-based rehabilitation (10) as these technologies offer flexibility in time and location and allow remote monitoring from the therapist (9). Furthermore, the recent COVID-19 pandemic has escalated this urgency to use home-based technologies to deliver the core components of rehabilitation as in-person services were discouraged from curbing the spread of the pandemic (11, 12).

Home-based upper limb (UL) rehabilitation refers to upper limb interventions conducted in the patient's home (permanent address, including other supported or sheltered home). The intervention is either self-directed or therapist-supervised and is conducted with or without technology. Technology-assisted interventions include virtual reality, telerehabilitation, robotics, interactive video games, wearable devices, transcranial direct current stimulation, brain–computer/machine interfaces, and electrical stimulation (10, 13). “No technology” interventions refer to mirror therapy, mental practice, music therapy, constraint-induced movement therapy (CIMT), bilateral upper limb training, task-specific training, and strength training (6).

Previous reviews and meta-analyses supported the effectiveness of home-based rehabilitation services in improving the patients' performance in their daily living activities (ADL), physical function, and quality of life (14–16). These reviews adopt a broad view of the effect of the home-based intervention on the stroke survivors' overall functional performance using outcome measures such as the Barthel index (BI) and functional independence measure (FIM). While contributing valuable knowledge on home-based stroke rehabilitation, the treatment effect on the stroke survivors' hemiplegic upper limb remained unclear.

The effectiveness of home-based upper limb rehabilitation interventions to promote the motor recovery of the hemiplegic upper limb among stroke survivors is supported by several previous studies (8, 17–19). Nevertheless, a collective evaluation of the available evidence in this area remained scarce. To our knowledge, there is one Cochrane review, undertaken in 2012 (20), which reviewed the effects of home-based therapy targeting upper limb recovery after stroke. Due to the lack of information available, only four RCT studies were included in the review. This Cochrane review (20) found that the effectiveness of a home-based upper limb rehabilitation was not superior to that of usual care. With insufficient high-quality evidence, the impact of home-based therapy programs for arm recovery in stroke survivors remained inconclusive (20). As this review was conducted a decade ago, the results of more recent studies were not evaluated.

Another more recent review by Da-Silva et al. (21) examined the literature on self-directed home-based upper limb interventions for the stroke population. This study discovered that the most effective home-based self-directed interventions are constraint-induced therapy, electrical stimulation, and no technology interventions. Nevertheless, the review by Da-Silva et al. (21) narrowed its scope to self-directed upper limb rehabilitation in the home setting. Self-directed intervention is conducted independently by the patients and carers without formal support or supervision by a healthcare professional (i.e., a therapist). Other forms of home-based upper limb interventions, such as those with direct or remote supervision by a therapist or healthcare professional, are not explored in this previous review (21).

More recent evidence and increased availability of advanced technologies in the home setting might significantly influence the evaluation of the updated evidence of upper limb rehabilitation in the home setting. The objectives of this review were to determine the effects of home-based upper limb interventions on improving hemiparetic upper limb function when compared to conventional therapy, placebo, or no intervention in stroke survivors and to identify the types of home-based interventions with optimal benefits to improve the hemiparetic upper limb function in the stroke survivors (see Supplementary Information 1: PICOS statement on review question). The finding of this review will aid researchers and clinicians by providing valuable insights into the updated evidence of the effects of home-based upper limb intervention in stroke rehabilitation and uncover critical elements for its successful implementation.

## Methods

### Search strategy

The Preferred Reporting Items for Systematic Reviews and Meta-analysis (PRISMA; 19) statement was used to structure this review. From January 2000 to September 2020, a systematic literature search was conducted in four electronic databases: Cochrane Library, MEDLINE, CINAHL, and Web of Science. The reference lists of relevant reviews were screened manually for additional relevant studies. The search strategy used a combination of the following keywords and their variations: “home-based,” “upper limb^*^,” “rehabilitation,” and “stroke^*^.” Variations of keyword combinations are shown in Supplementary Information 1.

### Selection criteria

This review followed the PICOS framework (22) for the inclusion of studies. Studies were considered for this review if they satisfied the following criteria.

#### Population (P)

Studies that involved adults (i.e., aged ≥ 18 years) with all stages of stroke; no restrictions were made concerning the type or localization of stroke.

#### Intervention (I)

Studies with one or more groups that received upper limb intervention in the home setting (or at least 80% of treatment carried out at home). Interventions targeted to improve upper limb function are self-directed, direct, or remote supervision from a therapist or healthcare professional, either technology-assisted or “no technology.”

#### Comparator(s)/Control (C)

Studies with a comparison group that received conventional therapy, placebo, or no intervention. Conventional treatment refers to the usual stroke rehabilitation care and interventions delivered in a hospital or clinic. If the studies compared two or more types of home-based interventions, the comparison group had to be a different type of intervention.

#### Outcomes (O)

Studies that measured outcomes on motor recovery of the hemiparetic upper limb, such as upper limb impairments, functional performance, and use in daily activities.

#### Study design (S)

Only randomized controlled trials (RCT) and randomized cross-over studies were included. Available studies published in English and have had a full-length publication were included.

Exclusion criteria of this review included: (1) qualitative studies, systematic, meta-analysis reviews, study protocols, and duplicates; (2) studies using non-stroke participants; and (3) studies using interventions that did not include upper limb training.

### Quality assessment and data extraction

Two independent reviewers (SFMT, CPF) screened for study eligibility based on titles and abstracts of references retrieved during the searches. The two reviewers (SFMT, CPF) independently reviewed the full text of pre-selected articles and agreed on the final set of articles through discussion. Two reviewers (SFMT, CPF) discussed and assessed the methodological quality of the included studies using the Physiotherapy Evidence Database (Pedro) scale, which is a valid and reliable measure of the methodological quality of randomized controlled trials (23). Any discrepancies were resolved *via* discussion with the third reviewer (KNKF). The Pedro scale allows the classification of high- and low-quality trials based on cut-off scores. A score of 6 and above on the Pedro scale was considered as “high” quality, and scores ranging from 4 to 5 were considered as “fair” quality, and any studies with a score below 4 were considered as “low” quality (24). The primary author-extracted data included: (1) author name; (2) sample size; (3) participants' details (i.e., age, gender, the onset of stroke); (4) intervention (i.e., content, dose, and duration); (5) clinical outcome measures; and (6) results (i.e., means, standard deviations, *p*-values).

### Data synthesis

A qualitative synthesis of the main results of the selected studies was presented in text and tables. This review included a narrative synthesis highlighting the methodological quality of the studies, types of intervention, participant characteristics, and outcome measures used.

In addition, a meta-analysis was carried out with the following data from the included studies to form a pooled estimate to report the effects of home-based interventions. Primary and secondary outcome measures that measured upper limb function were identified in each study and considered for the meta-analysis if data on mean scores and standard deviation (SDs) were available. All outcome measures were analyzed as continuous data using the means and SDs (25). If the studies reported outcome data as medians and interquartile ranges (IQR), the medians and IQR were converted into means and SDs using the formula developed by Hozo and colleagues (26). Most outcome measures in the included studies had rated improvement by a gain score. If a reduced outcome score indicated improvement (i.e., a decrease in time taken to perform a task), the scale direction was aligned with others by multiplying the score by −1 (25). For studies with a cross-over design, only the first phase data (before cross-over) were included in the analysis to prevent any possible learning or carryover effects that would contaminate the data (21). The mean change from baseline was used to compare control and intervention groups (25). For studies that did not report the mean change score and SD but provided pre and post/follow-up scores, the Open Meta-Analyst software (27) was used to calculate the mean change scores. A pooled mean difference (MD) estimate with 95% confidence (CI) was calculated if the studies used the same outcome measure.

Regarding studies that used different outcome measures deemed comparable, standardized mean difference (SMD) with 95% CI was calculated (25). Publication bias was evaluated graphically using funnel plots (28). Egger's linear regression test (29) was used to analyze five studies and above to assess publication bias in the funnel plot (30).

The heterogeneity of the selected studies was assessed using the *I*^2^ statistic; if *I*^2^ was >50% with a significant *p* < 0.1, the studies were considered heterogeneous (25), and a random model effect was used. A fixed model effect was used to pool study results with low heterogeneity with *I*^2^ ≤ 50% (25). In the case of high heterogeneity and significant publication bias, a sensitivity analysis was conducted on the included studies to confirm these effects after adjusting the included data (25, 28). Procedures related to data pooling in the meta-analysis were carried out in Review Manager 5.3 (31). Comprehensive Meta-analysis 3.0 software (32) was used to analyze publication bias (i.e., Egger regression test).

## Results

### Study selection

**Figure 1:**
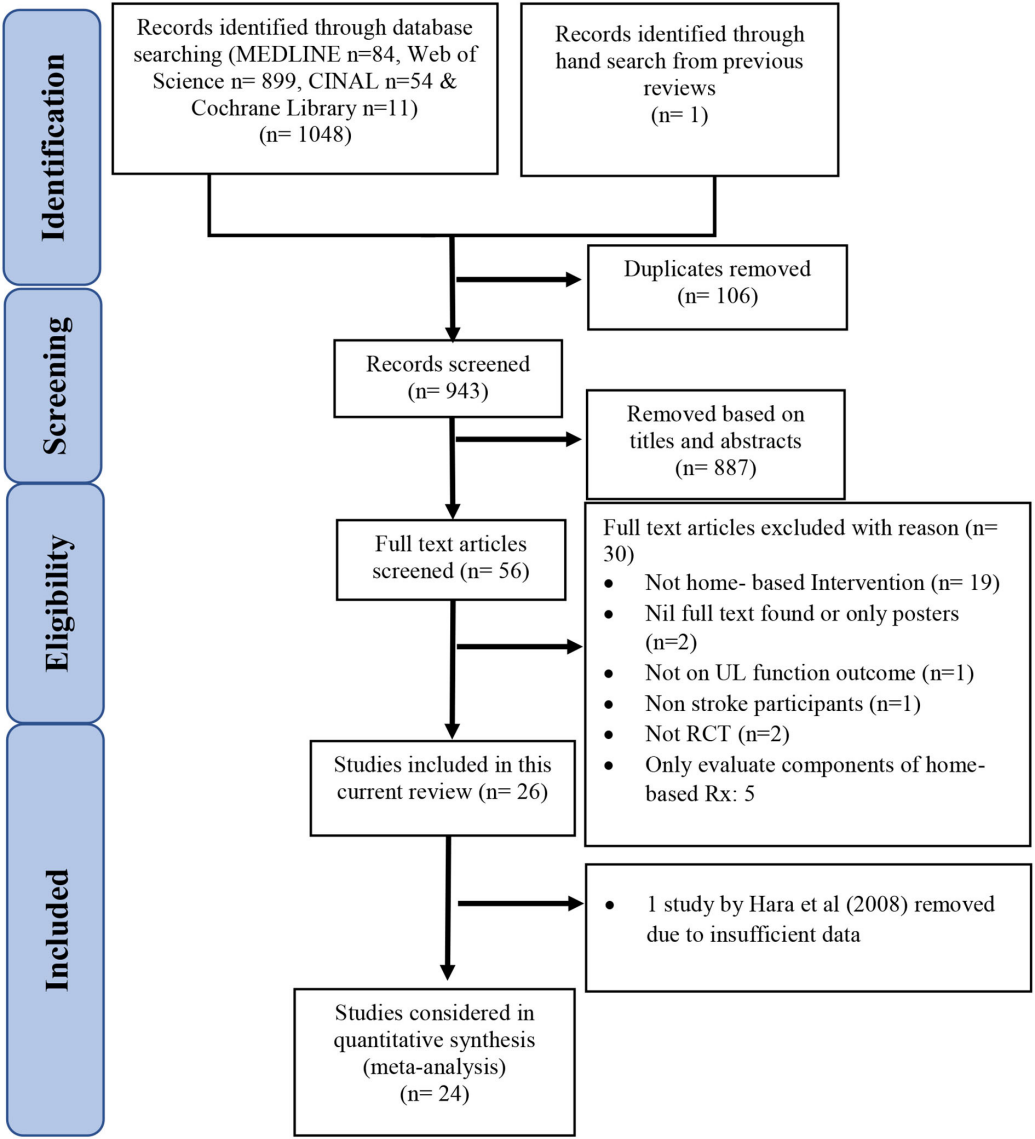
PRISMA diagram.

The PRISMA diagram (33) in Figure 1 summarizes the literature search results. The initial search from the four databases yielded 1,049 articles (Cochrane Library *n* = 11; CINAL *n* = 54; MEDLINE *n* = 84; Web of Science *n* = 899, hand search from references of relevant reviews *n* = 1; Supplementary Information 1). After removing 106 duplicates, 943 articles were screened. After screening through the titles and abstracts, 887 articles were excluded. Fifty-six articles were obtained as full texts for further review by the two reviewers (SFMT, CPF). Of these articles, 26 studies were selected. The characteristics of the remaining 30 excluded studies are shown in Supplementary Information 2.

### Characteristics of studies and participants

A total of 26 randomized studies were selected. Table 1 provides an overview of the chosen studies, including 21 randomized controlled trials (RCTs) and five randomized cross-over trials. All the selected studies were rated as fair to high quality with a mean score of 6.6 ± 1.2 (ranging from 4 to 8) on the Pedro Scale. Table 2 details the individual Pedro score of each study. The total number of participants in this review was 1,428, with the sample size ranging from 12 to 235. The mean age of the participants ranged from 52.3 to 69.4 years. The average time since stroke onset reported in the studies was 23.5 ± 21.2 months. One study (9) did not report the details of stroke onset in their participants but merely mentioned that their participants were in the acute phase of the stroke.

**Table 1:** Characteristics of included studies.

**Study**	***n* (E/C)**	**Age (yr)**	**Time since stroke**	**Primary outcome measures**	**Experiment**	**Control**	**Therapy dose**	**Results**
Adie et al. (34)	117/118	E: 66.8 ± 14.6 C: 68 ± 11.9	E: 57.3 ± 48.3 (d) C: 56.3 ± 50.1 (d)	ARAT	Home-based Wii grp	Home exercise handout	45 min, daily for 6 wks	No between grp difference (MD: −1.7, 95% CI −3.9–0.5, *p* = 0.12) on ARAT score to improve UL function
Ballester et al. (35)	17/18	E: 65.1 ± 10.3 C: 61.8 ± 12.9	E: 1,073.4 ± 767.7 (d) C: 798.1 ± 421.8 (d)	FM, CAHAI	Home-Based VR	Home-based OT	E: 26 min 40 s, 1–3 times/d, 5 d/wk, 3 wks C: 20 min, 1–3 times/d, 5 d/wk, 3 wks	VR was more effective to improve UL function measured by CAHAI scale [1.53 (2.4), *p* = 0.01] than home-based OT
Barzel et al. (36)	85/71	E: 62.6 ± 13.7 C: 65.3 ± 13.7	E: 56.6 ± 47.4 (mo) C: 45.7 ± 57.7 (mo)	MAL WFMT	Home-Based CIMT	NDT clinic-based	E: 50–60 min, 5 times/5 wks + 40 h in 20 d of self-practice C: 25–30 min, 10 times/5 wks or 50–60 min, 5 times/5 wks	Home-based CIMT grp improved more in MAL scores (MD: 0.26, 95% CI 0.05–0.46, *p* = 0.016) than NDT grp
Choudhury et al. (37)	32/32	E: 51 ± 12.1 C1: 53 ± 9.9 C2: 53.0 ± 10.6	E: 55 ± 142 (mo) C1: 43 ± 94 (mo) C2: 30 ± 29 (mo)	ARAT, MA S, power and pinch strength, maximum force at wrist joint	Paired stim	C1: Random stim C2: Usual care	4 h/d over 4 wks	Paired stim grp improved more ARAT (median baseline: 7.5, week 8: 11.5, *p* = 0.019) than the other two trainings
Cramer et al. (38)	62/62	E: 62 ± 14 C: 60 ± 13	E: 132 ± 65 (d) C: 129 ± 59 (d)	FM	Home-Based telerehab	Clinic	18 supervised and 18 unsupervised 70 min sessions, over 4 wks.; 5 min/d ×3 times of stroke education	No between grp difference on FM score (0.06, 95% CI −2.14–2.26, *p* = 0.96) was found
dos Santos-Fontes et al. (39)	10/10	E: 52.2 ± 11.1 C: 59.1 ± 11.1	E: 3.8 ± 4.5 (yr) C: 3.3 ± 2.1 (yr)	JTT Compliance rate	Home-Based RPSS stim	Sham	2 h of stim daily before motor training, over 4 wks Motor training for 15 min, 2 times/d in 4 wks at home	Electrical stim grp improved more in JTT performance than sham grp (14.3%, CI = 1.06–25.6%)
Duncan et al. (17)	50/50	E: 68.5 ± 9 C: 70.2 ± 11.4	E: 77.5 ± 28.7 (d) C: 73.5 ± 27.1 (d)	OPS, FM, Grip strength, WMFT	Home therapeutic exercise	Usual care	E: 36 sessions, 90 min over 12–14 wks C: not specific	The overall effect of therapeutic exercise had greater gain than usual care (Wilk's λ = 0.64, *p* = 0.0056)
Emmerson et al. (40)	30/32	E: 68 ± 15 C: 63 ± 18	E: 122 (77–193; d, median) C: 133 (58–228; d, median)	Adherence rate WMFT	Home-Based iPad grp	Home exercise handout	1–2 times/d with no of exercises varied per d, for 4 wks	No between grp difference (MD: 0.02s, 95% CI −0.1–0.1) on WMFT log-transformed time to improve UL function
Hara et al. (41)	10/10	E: 56 C: 60.5	E: 13 (mo) C: 13 (mo)	SIAS, ROM, MAS, 10-CMT, & 9-HPT	Home-Based FES grp	Clinic	E: 30 min, 5 d/wk for first 10 days, then 1 h/session, 5 d/wk for 5 mo C:40 min, once/wk for 5 mo	Home-based FES was more effective to improve UL function than outpatient rehab (10-CMT: *F* = 18.72, *p* < 0.01)
Hsieh et al. (18)	12/12	E: 53.2 ± 19.2 C: 56.4 ± 18	E: 15.9 ± 13 (mo) C: 13.7 ± 11 (mo)	FM, BBT, Revised NSA, MAL, 10 m walk, sit-to-stand test, COPM, EuroQoL-5D	Home-Based MT	MT in clinic	75–105 min, for 12 sessions over 4 wks	Home-based MT grp improved more than clinic MT on MAL (*p* = 0.01)
Kimberly et al. (42)	8/8	E: 58.4 C: 62.8	E: 24.6 (mo) C: 38.5 (mo)	Grip strength, BBT, MAL, JTT, Isometric finger extension strength	Home-Based NMES	Sham	3–6 h, for 10 d over 3 wks	Home-based NMES improved arm function more than sham [BBT: *t*_(7)_ = 2.06, *p* = 0.039; JTT: *t*_(7)_ = 3.82, *p* = 0.003; MAL-AOU: *t*_(7)_ = 7.6, *p* < 0.001; MAL-QOM: *t*_(7)_ = 3.82, *p* = 0.003]
Mortenson et al. (43)	8/8	E: 65.5 C: 60.8	E: 32 (mo) C: 28.8 (mo)	JTT, grip strength	Home-Based transcranial stim	Home-Based CT	30 min per session, 5 times	Both groups improved in JTT over time (*p* < 0.01). Anodal grp improved more in grip strength than sham (*p* = 0.025)
Michielsen et al. (44)	20/20	E: 55.3 ± 12 C: 58.7 ± 13.5	E: 4.7 ± 3.6 (yr) C: 4.5 ± 2.6 (yr)	FM, Grip strength, Tardieu scale, VAS, ARAT, ABILHAND, Stroke-ULAM, EQ-5D	Home-Based MT	Home-Based bilateral UL training	1 h per session, 5 times/wk at home, 1 time/wk at center over 6 wks	MT grp improved more in FM than bilateral training grp after Rx (3.6 ± 1.5, *p* < 0.05)
Nijenhuis et al. (45)	9/10	E: 58 (48–65) C: 62 (54–70)	E: 11 (10–26; mo) C: 12 (10–30; mo)	IMI, FM, grip strength, MAL, ARAT, BBT, SIS	Home-Based robotic	Home-Based CT	30 min per session, 5 times/wk over 6 wks at home	CT grp reported higher training duration (189 vs. 118 min per wk, *p* = 0.025). No between groups difference in UL outcomes (*p* ≥ 0.165)
Piron et al. (46)	18/18	E: 66.0 ± 7.9 C: 64.4 ± 7.9	E: 14.7 ± 6.6 C: 11.9 ± 3.7	FM, ABILHAND scale, Ashworth scale	Home-based telerehab	Clinic	1 h per session, 5 times/wk over 4 wks at home	Telerehab grp improved more in FM (53.6 ± 7.7) than clinic (49.5 ± 4.8), *p* < 0.05
Saadatnia et al. (9)	20/20	E: 62 ± 12.4 C: 66 ± 10.3	Nil data	BI, FM, MRS	Home-Based video exercise	Usual care (in clinic)	E: 1 h per session, 2 times/d, daily over 12 wks at home + usual care C: usual care	Video exercise grp improved more in BI, FM, and MRS score than usual care grp (*p* < 0.001)
Standen et al. (47)	17/10	E: 59 ± 12 C: 63 ± 12	E: 22 (16, 59.5; mo) C: 12 (7.75, 20.25; mo)	WMFT, 9-HPT, MAL, Nottingham extended activities of daily living	Home-Based Nintendo VR	No Rx	E: 20 min per session, 3 times/wk over 8 wks C: nil	VR grp improved more than control grp in WMFT (*r* = 0.51, *p* < 0.05) at midpoint and MAL-AOU (*r* = 2.26, *p* < 0.05) at final point
Street et al. (48)	6/6	E: 53.2 ± 21.9 C: 67.6 ± 18.3	E: 19 (mo) C: 13.8 (mo)	ARAT, 9-HPT	Home-Based (TIMP)	No treatment	E: 20–30 min per session, 2 times/wk over 6 wks C: nil	No between grp difference in overall ARAT score 1.313 (SE:0.674, 95%CI: −0.073–2.698) and 9-HPT 0.169 (SE:0.823, 95%CI: −1.53–1.87)
Stinear et al. (49)	16/16	E: 57.9 (38–78) C: 52.6 (25–73)	E: 28.8 (6–144; mo) C: 20.3 (6–73; mo)	FM, NIHSS, grip strength	Home-Based (APBT)	Self-Directed task training	10–15 min per session, 3 times/wk over 4 wks	APBT grp improved more UL function (*p* < 0.025) than control grp
Sullivan et al. (50)	20/18	E: 61.6 ± SD (37–88) C: 59.5 ± SD (41–85)	E: 7.7 ± SD (1–29; yr) C: 6.6 ± SD (3–14; yr)	FM, AMAT	Home-Based sensory electrical stimulation (SES)	Sham	30 min, 2 times/d, 5 d/wk over 4 wks	No between grp differences but SES grp improved more on AMAT median time (*p* = 0.003, 95% CI:−1.4304, −6.365, effect size: 0.84) after Rx
Tariah et al. (51)	10/8	E: 54.8 ± 10.9 C: 60.6 ± 4.9	E: 9.2 ± 5.8 (mo) C: 9.6 ± 4 (mo)	WMFT	Home-Based CIMT	Outpatient NDT	2 h/d, 7 d/wk over 8 wks	CIMT grp improved more in WMFT-FAS [*F*_(1, 15)_ = 12.68, *p* = 0.003] as compared to NDT grp
Turton et al. (52)	24/23	E: 66 (54.3, 75.1; median; IQR) C: 66.1 (57.6, 76.5; median; IQR)	E: 111.5 (82, 241) (d) C: 135 (103, 171) (d)	ARAT, WMFT	Home-Based reach-to-grasp (RTG)	Usual care	E:14 visits, 1 h/visit over 6 weeks + 56 h of self-practice C: not specific	RTG grp improved 6 points for median score of ARAT after Rx but not the usual care grp
Wei et al. (8)	32/25/27	E: 59.2 ± 11.3 C1: 60.4 ± 10.4 C2: 63.1 ± 10.3	E: 47.8 ± 21.9 (d) C1: 61.1 ± 41.3 (d) C2: 53.7 ± 41.2 (d)	FM, ARAT, BBT	Home-Based wearable device	C1: sham C2: usual care	E & C1: 3 h/d,7 d/wk over 4 wks C2: not specific	Wearable grp improved more in ARAT score than sham (MD = 6.283, 95% CI 0.812–11.752, *p* = 0.019) and control (MD = 5.767, 95% CI 0.299–11.235, *p* = 0.035)
Wolf et al. (53)	51/48	E: 59.1 ± 14.1 C: 54.7 ± 12.2	E: 115.5 ± 53.1 (d) C: 127.1 ± 46.2 (d)	ARAT	Home-Based robotic	Home exercise handout	3 h/d, 5 d/wk over 8 wks	Control group improved more in WMFT than robotic grp (*p* =0.012)
Zondervan et al. (54)	8/8	E: 61 ± 17 C: 54 ± 14	E: 39 ± 46 (mo) C: 24 ± 8 (mo)	FM	Home-Based Resonating arm exercise (RAE)	Conventional therapy	3 h/3 sessions/wk over 3 wks	Both groups improved in FM (*p* < 0.05) after Rx. RAE grp improved more in distal FM than CT (*p* = 0.02)
Zondervan et al. (55)	9/8	E: 60 (bib45–74) C: 59 (35–74)	E: 5.33 ± 4.14 (y) C: 3.17 ± 1.66 (y)	BBT, ARAT, MAL, & 9-hole peg test	Home-Based music glove (VR)	Home-Based task-specific training	3 h/wk over at least 3 sessions/wk for 3 wks	No between grp difference in ARAT. VR grp improved more in both subscales of MAL (*p* = 0.007, *p* = 0.04)

AMAT, Arm Motor Ability Test; ARAT, Action Research Arm Test; APBT, active passive bilateral training; BI, Barthel Index; BBT, Box and Block Test; C, control; COPM, Canadian Occupational Performance Measure; CAHAI, Chedoke Arm and Hand Inventory; CI, confidence index; CT, conventional therapy; E, experiment; EQ-5D-3L; d, days; FIM, functional independence measure; FM, Fugl-Meyer; h, hours; HEP, home exercise programme; IMI, Intrinsic Motivation Inventory; JTT, Jebsen–Taylor Test; MAL, Motor Activity Log; mo, months; MMT, manual muscle testing; MRS, Modified Rankin Scale; NIHSS, National Institute of Health Stroke Scale; 9-HPT, Nine hole Peg Test; OPS, Orpington Prognostic Scale; MT, mirror therapy; RNSA, Revised Nottingham Sensory Assessment; RCT, randomized controlled trial; RPSS, repetitive peripheral sensory; Rx, Treatment; SIS, Stroke Impact Scale; SIAS, Stroke Impairment Assessment Scale; SE, standard error; TEMPA, the upper extremity performance test; TIMP, therapeutic instrumental music performance;10-CMT, 10-cup-moving test; VAS, visual analog scale for pain; WMFT, Wolf Motor Function Test; WMFT-FAS, Wolf Motor Function Test -functional ability score; wk, weeks; yr, years; Rx, treatment.

**Table 2:** Methodological quality of studies.

**Studies**	**Pedro scale**												
	**1**	**2**	**3**	**4**	**5**	**6**	**7**	**8**	**9**	**10**	**11**	**Total**	**Type**
Adie et al. (34)	Yes	1	1	1	0	0	1	1	1	1	1	8	H
Ballaster et al. (35)	Yes	1	0	1	0	0	0	1	0	1	1	5	F
Barzel et al. (36)	Yes	1	1	1	0	0	1	1	1	1	1	8	H
Choudhury et al. (37)	Yes	1	1	1	0	0	1	1	1	1	1	8	H
Cramer et al. (38)	Yes	1	1	1	0	0	1	1	1	1	1	8	H
Dos-Sanrtose-fontes et al. (39)	Yes	1	1	1	0	0	1	1	1	1	1	8	H
Duncan et al. (17)	Yes	1	1	1	0	0	1	1	1	1	1	8	H
Emmerson et al. (40)	Yes	1	1	1	0	0	1	1	0	1	1	7	H
Hara et al. (41)	Yes	1	0	0	0	0	1	1	0	1	1	5	F
Hsieh et al. (18)	Yes	1	0	1	0	0	1	0	0	1	1	5	F
Kimberly et al. (42)	Yes	1	0	1	1	0	1	1	0	0	1	6	H
MIchielsen et al. (44)	Yes	1	1	1	0	0	1	1	1	1	1	8	H
Mortensen at al. (43)	Yes	1	1	1	1	0	1	1	0	1	1	8	H
Nijenhuis et al. (45)	Yes	1	1	1	0	0	0	1	0	1	1	6	H
Prion et al. (46)	No	1	1	1	0	0	1	1	0	1	1	7	H
Saadatnia et al. (9)	Yes	1	0	1	0	0	0	0	0	1	1	5	F
Standen et al. (47)	No	1	1	1	0	0	1	0	0	1	1	6	H
Stinear et al. (49)	Yes	1	0	1	0	0	1	0	0	1	1	5	F
Street et al. (48)	Yes	1	0	1	0	0	1	1	0	1	1	6	H
Sullivan et al. (50)	Yes	1	0	1	0	0	1	1	1	1	1	7	H
Tariah et al. (51)	Yes	1	0	1	0	0	1	1	0	1	1	6	H
Turton et al. (52)	Yes	1	1	1	0	0	1	1	1	0	1	7	H
Wei et al. (8)	Yes	1	0	1	0	0	1	1	1	1	1	7	H
Wolf et al. (53)	Yes	1	0	1	0	0	1	1	1	1	1	7	H
Zondervan et al. (54)	Yes	0	0	1	0	0	1	1	1	1	1	6	H
Zondervan et al. (55)	Yes	1	0	1	0	0	1	1	0	1	1	6	H

1, eligibility criteria; 2, random allocation; 3, concealed allocation; 4, baseline comparability; 5, blind participants; 6, blind therapist; 7, blind assessor; 8, adequate follow-up; 9, intention to treat; 10, between group comparison; 11, point estimate variability. Quality: High Quality (H), Fair Quality (F), Low Quality (L).

### Types of home-based upper limb interventions

This review included studies that made three types of comparisons: (1) studies that compared the home-based upper limb intervention to conventional therapy conducted in a clinic or hospital (clinic-based therapy), (2) studies that compared the home-based upper limb intervention to no treatment, and (3) studies that compared two different types of home-based interventions. Thirteen studies (8, 9, 17, 18, 36–38, 41, 46–48, 51, 52) compared home-based intervention to either clinic-based therapy or no intervention. Among these studies, 11 (8, 9, 17, 18, 36–38, 41, 46, 51, 52) used a control group that had undergone clinic-based therapy, and two (47, 48) had control groups that did not receive any treatment. The remaining 13 (34, 35, 39, 40, 42–45, 49, 50, 53–55) studies compared two types of home-based interventions. Of which, 10 studies (34, 35, 39, 40, 42, 43, 45, 50, 53, 55) compared technology-assisted home-based upper limb intervention to “no technology” interventions, while another three studies compared two kinds of “no technology” home-based interventions (44, 49, 54).

Eighteen studies (8, 9, 34, 35, 37–43, 45–48, 50, 53, 55) used technology-assisted home-based upper limb interventions in their experimental groups to examine the treatment effects on hemiplegic upper limb recovery. In these studies, the technology-assisted interventions used were interactive video games (on devices such as Wii, iPad, Kinect), virtual reality, electrical stimulation (including transcranial stimulation), robotics, telerehabilitation, and wearable devices. Most of these 18 studies (*n* = 15) adopted either self-directed or remote supervision by a therapist in delivering the interventions (8, 34, 35, 37–42, 45–47, 50, 53, 55). Three studies (9, 43, 48) used direct supervision or a hybrid model. Table 3 presents the types of home-based upper limb interventions and the mode of delivery.

**Table 3:** Types of interventions and mode of delivery.

**Studies**	**Types of “experiment” intervention**	**Classification of “experimented” intervention**	**Mode of delivery**
**Comparison 1: Home-Based UL therapy to clinic-based therapy**			
Barzel et al. (36)	Home-CIMT	“No” tech	Exp grp: Hybrid Self-directed & Direct supervised Con grp: Direct supervised
Choudhury et al. (37)	Electrical stimulation	Tech-Assisted	Exp grp: Self-directed Con grp: Direct supervised
Cramer et al. (38)	Telerehabilitation	Tech-Assisted	Exp grp: Remote supervised Con grp: Direct supervised
Duncan et al. (17)	Therapeutic exercise	“No” tech	Both grps: Direct supervised
Hara et al. (41)	Electrical stimulation	Tech-Assisted	Exp grp: Self-directed Con grp: Direct supervised
Hsieh et al. (18)	Mirror therapy	“No” tech	Both grps: Direct supervised
Piron et al. (46)	Telerehabilitation	Tech-Assisted	Exp grp: Remote supervised Con grp: Direct supervised
Saadatnia et al. (9)	Virtual reality	Tech-Assisted	Exp grp: Hybrid Self-directed & Direct supervised Con grp: Direct supervised
Tariah et al. (51)	Home-CIMT	“No” tech	Exp grp: Hybrid Self-directed & Direct supervised Con grp: Direct supervised
Turton et al. (52)	Task-specific training	“No” tech	Exp grp: Hybrid Self-directed & Direct supervised Con grp: Direct supervised
Wei et al. (8)	Wearable device training	Tech-Assisted	Exp grp: Self-directed Con grp: Direct supervised
**Comparison 2: Home-based UL therapy to no intervention**			
Standen et al. (47)	Virtual reality	Tech-Assisted	Exp grp: Self-directed Con grp: NA
Street et al. (48)	Music therapy	Tech-Assisted	Exp grp: Direct supervised Con grp: NA
**Comparison 3: Home-Based technology to “no tech” intervention**			
Adie et al. (34)	Virtual reality	Tech-Assisted	Both grps: Self-directed
Ballester et al. (35)	Virtual reality	Tech-Assisted	Both grps: Self-directed
dos Santos-Fontes et al. (39)	Electrical stimulation	Tech-Assisted	Both grps: Self-directed
Emmerson et al. (40)	Virtual reality	Tech-Assisted	Exp grp: Remote supervised Con grp: Self-directed
Kimberly et al. (42)	Electrical stimulation	Tech-Assisted	Both grps: Self-directed
Mortenson et al. (43)	Electrical stimulation	Tech-Assisted	Both grps: Direct supervised
Nijenhuis et al. (45)	Robotics	Tech-Assisted	Both grps: Self-directed
Sullivan et al. (50)	Electrical stimulation	Tech-Assisted	Both grps: Self-directed
Wolf et al. (53)	Robotics	Tech-Assisted	Both grps: Self-directed
Zondervan et al. (55)	Virtual reality	Tech-Assisted	Both grps: Self-directed
**Comparison 4: Two types of “no technology” home-based interventions**			
Michielsen et al. (44)	Mirror therapy	“No” tech	Both grps: Self-directed
Stinear et al. (49)	Mechanical device	“No” tech	Both grps: Self-directed
Zondervan et al. (54)	Mechanical device	“No” tech	Both grps: Self-directed

Con, control; Exp, experiment; grp, group; tech, technology; NA, not applicable.

The remaining eight studies (17, 18, 36, 44, 49, 51, 52, 54) used “no technology” interventions in the home setting. These interventions included home-based constraint-induced movement therapy (HOME-CIMT), task-specific training, therapeutic exercise, mirror therapy (MT), and mechanical device training. The HOME-CIMT used in the two included studies (36, 51) was different from the traditional CIMT in which all training was conducted solely at the participants' homes and not in the clinic. Three-quarters of these “no” technology home-based interventions (17, 18, 36, 44, 51, 52) involved direct contact with the therapists, with only two studies using a self-directed mode (49, 54).

### Outcome measures

This review primarily focused on upper limb motor and functional use outcomes. The outcome measures varied across the studies. Eighteen studies (8, 9, 17, 18, 35, 37, 38, 41–46, 49–51, 53, 54) used outcome measures that measured upper limb impairments. The most popular outcome measures used were the Fugl-Meyer Assessment-Upper Extremity subscore (FMA-UE; *n* = 14), followed by grip strength (*n* = 8) and ROM (*n* = 2). Twenty-three studies (8, 17, 18, 34–45, 47, 48, 50–55) measured intervention effects using outcome measures that assessed arm function. The commonly reported outcome measures were the Action Research Arm Test (ARAT; *n* = 9), Wolf Motor Function Test (WMFT; *n* = 7), Box and Block Test (BBT; *n* = 7), Nine-hole Peg Test (9-HPT; *n* = 5), and Jebsen–Taylor Test (JTT; *n* = 3).

This review considered the participant's perception of the affected arm use in daily activities as one of the focused outcomes. Though it is commonly assumed that improvements in the upper limb capacity as measured by standardized upper limb assessments would translate into improved use of the affected arm in daily activities, Waddle et al. (56) highlighted that it is not the case. Eleven studies (8, 18, 36, 42, 45, 47, 50–52, 54, 55) used the Motor Activity Log (MAL) to assess the participant's perception of the affected arm. The MAL is a self-reported questionnaire to assess how often and well the patients used their affected arm daily (57). It consisted of two subscales: the amount of use (MAL-AOU) and quality of movement (MAL-QOM) of the paretic arm.

### Effects of interventions

A meta-analysis was conducted to examine the clinical effects of home-based upper limb interventions. This review included three categories of studies: (1) studies that compared the home-based upper limb interventions to clinic-based therapy, (2) studies that compared two forms of home-based upper limb interventions (technology-assisted and “no technology”), and (3) studies that compared home-based upper limb interventions to no intervention. To address the review's objectives, comparisons were made on the effects of the studies in these three categories. Funnel plots of the meta-analysis are shown in Supplementary Information 3.

#### Home-based UL intervention vs. clinic-based therapy

**Figure 2:**
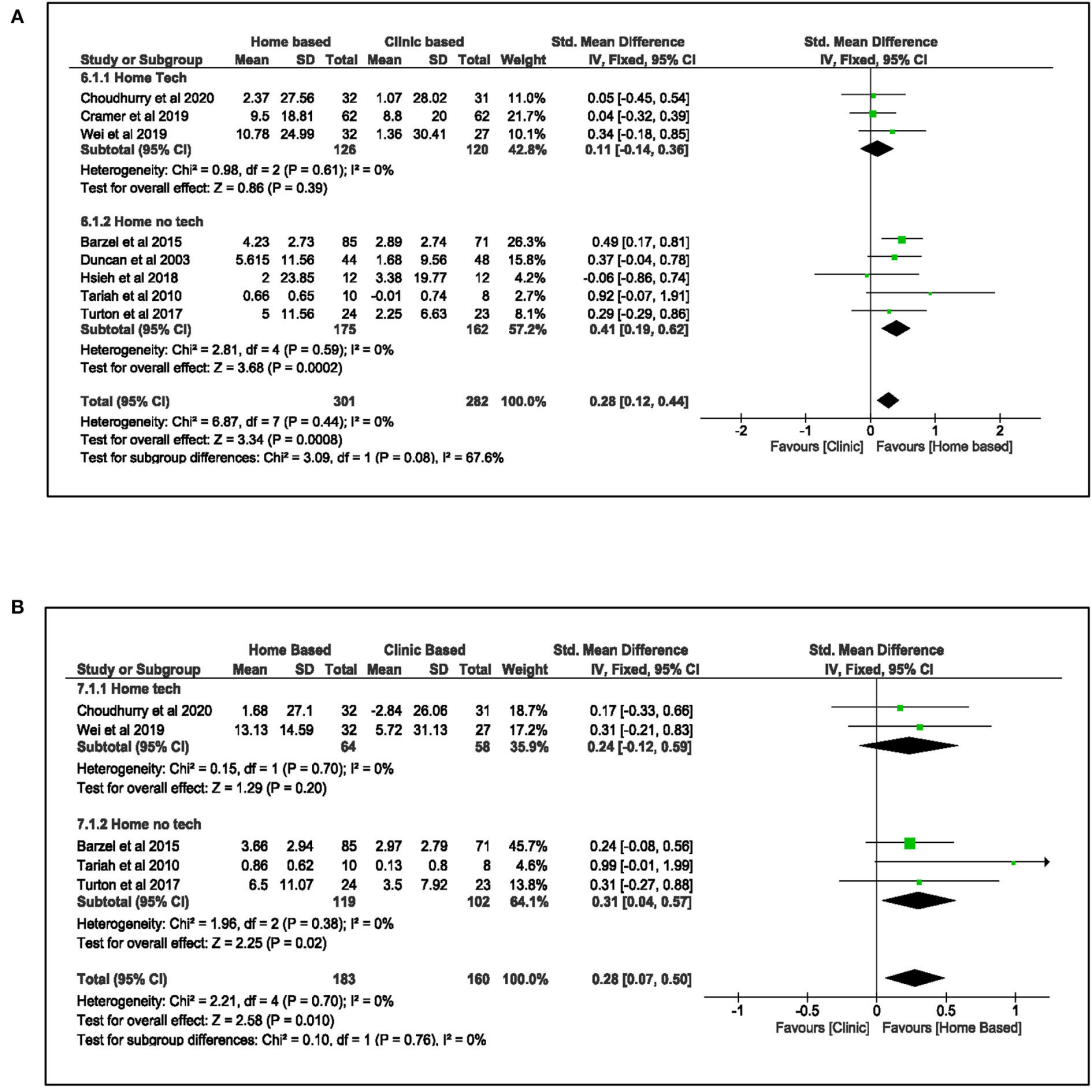
Comparison of the effect of home-based intervention and conventional therapy on UL function **(A)** standardized mean difference (SMD) immediately after the treatment. **(B)** Standardized mean difference (SMD) at follow-up.

A pooled meta-analysis (Figure 2) involving eight studies (8, 17, 18, 36–38, 51, 52) was carried out to examine the effect of home-based upper limb interventions on the function of the upper limb when compared to clinic-based therapy immediately after treatment and at follow-up. In these eight studies (8, 17, 18, 36–38, 51, 52), three studies used home-based technology-assisted interventions such as electrical stimulation, wearable device, and telerehabilitation (8, 37, 38), and these interventions were either self-directed or remotely supervised by a therapist. The other five studies (17, 18, 36, 51, 52) used “no technology” interventions such as HOME-CIMT, mirror therapy, therapeutic exercises, and goal-oriented task-specific training. All these “no technology” interventions required direct contact with the therapist. A mixture of upper limb outcome measures was used in these eight studies. Three studies used ARAT (8, 37, 52), another three used WMFT (17, 36, 51), and two studies used Box and Block Test (18, 38).

The pooled effects from the meta-analysis demonstrated that home-based upper limb intervention improved the hemiplegic upper limb function more significantly than clinic-based therapy [SMD: 0.28, 95% CI (0.12, 0.44), *I*^2^ = 0%, *p* < 0.001, fixed effect model]. The funnel plot (see Figure S2A in Supplementary Information 3) showed no publication bias supported by Egger's test (β: 0.04, SE: 1.24, *p* = 0.98). Subgroup analysis revealed that studies that used “no technology” home-based interventions contributed to the favorable pooled result. These studies (17, 18, 36, 51, 52) indicated a statistically significant benefit over clinic-based therapy to improve UL function immediately after treatment [SMD: 0.41, 95%CI (0.19, 0.62), *I*^2^ = 0%, *p* < 0.001, fixed effect model]. In contrary, studies that used home-based technology-assisted upper limb intervention (8, 37, 38) did not show similar effects [SMD: 0.11, 95% CI (−0.14, 0.36), *I*^2^ = 0%, *p* = 0.39, fixed effect model].

The pooled results from five studies (8, 36, 37, 51, 52) that measured the effects of home-based upper limb intervention at follow-up demonstrated that the improvements in upper limb function were sustained [SMD: 0.28, 95% CI (0.07, 0.50), *I*^2^ = 0%, *p* = 0.01, fixed effect model] with no publication bias (Egger's test: β: 1.56, SE: 0.72, *p* = 0.12). Similarly, further analysis showed that studies (36, 51, 52) that used “no technology” interventions were the main contributor to this effect [SMD: 0.31, 95% CI (0.04, 0.57), *I*^2^ = 0%, *p* = 0.02, fixed effect model].

**Figure 3:**
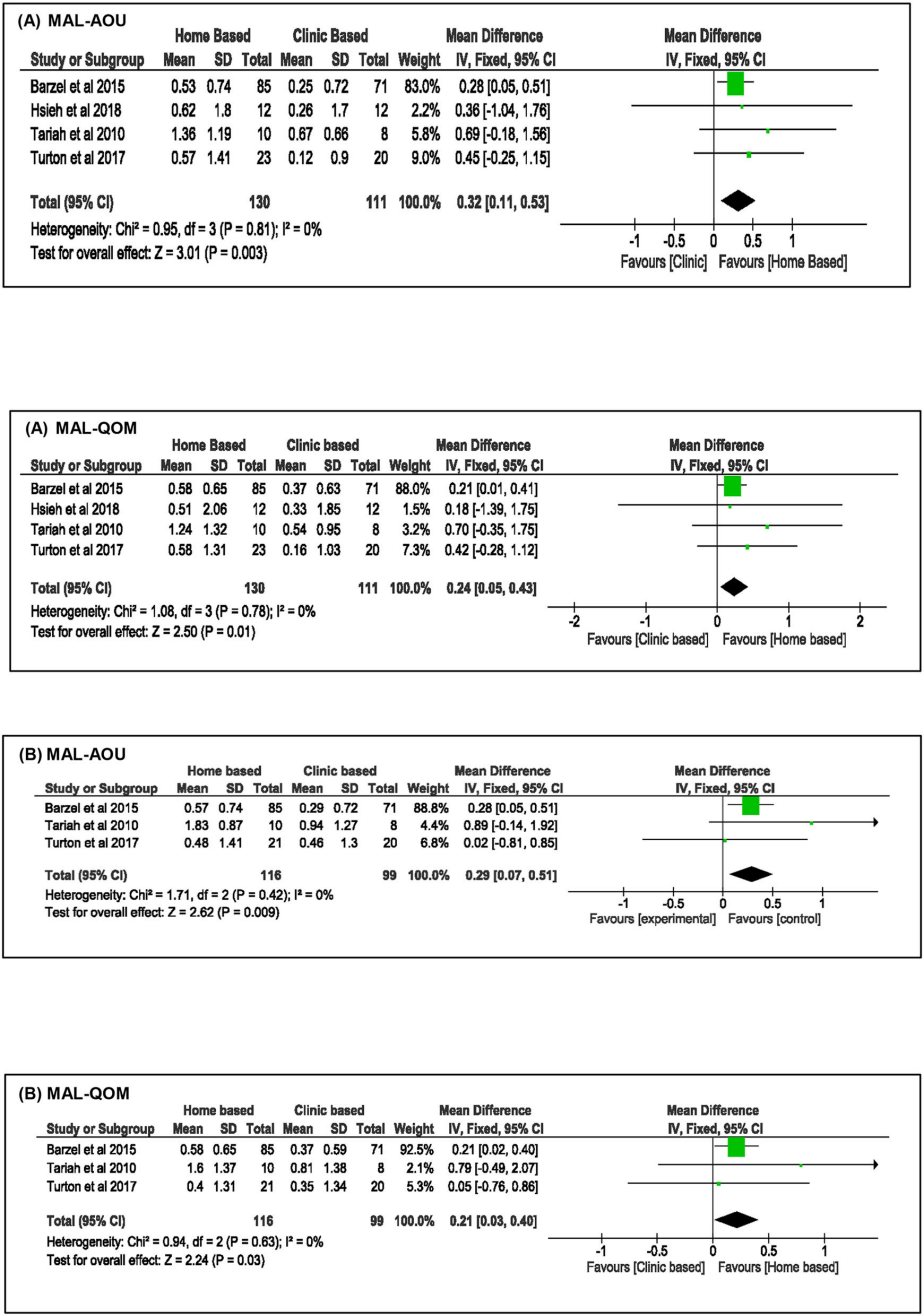
Comparison of the effect of home-based intervention and conventional therapy on MAL outcomes **(A)** mean difference (MD) immediately after the treatment. **(B)** Mean difference (MD) at follow-up.

Besides the improvements in upper limb function, the effects of home-based upper limb interventions on the participants' perceived use of their paretic arm in daily routine were analyzed using the MAL outcomes. Meta-analysis of four studies (18, 36, 51, 52) (Figure 3) demonstrated that the home-based intervention group improved more than the clinic-based intervention group in the MAL scores: MAL-AOU [MD: 0.32, 95% CI (0.11, 0.53), *I*^2^ = 0%, *p* = 0.003, fixed effect model] and MAL-QOM [MD: 0.24, 95% CI (0.05, 0.43), *I*^2^ = 0%, *p* = 0.01, fixed model]. This positive effect was sustained at follow-up: MAL-AOU [MD: 0.29, 95% CI (0.07, 0.51), *I*^2^ = 0%, *p* = 0.009, fixed effect model], and MAL-QOM [MD: 0.21, 95% CI (0.03, 0.40), *I*^2^ = 0%, *p* = 0.03, fixed effect model].

#### Home-based technology-assisted intervention vs. “no technology” intervention

**Figure 4:**
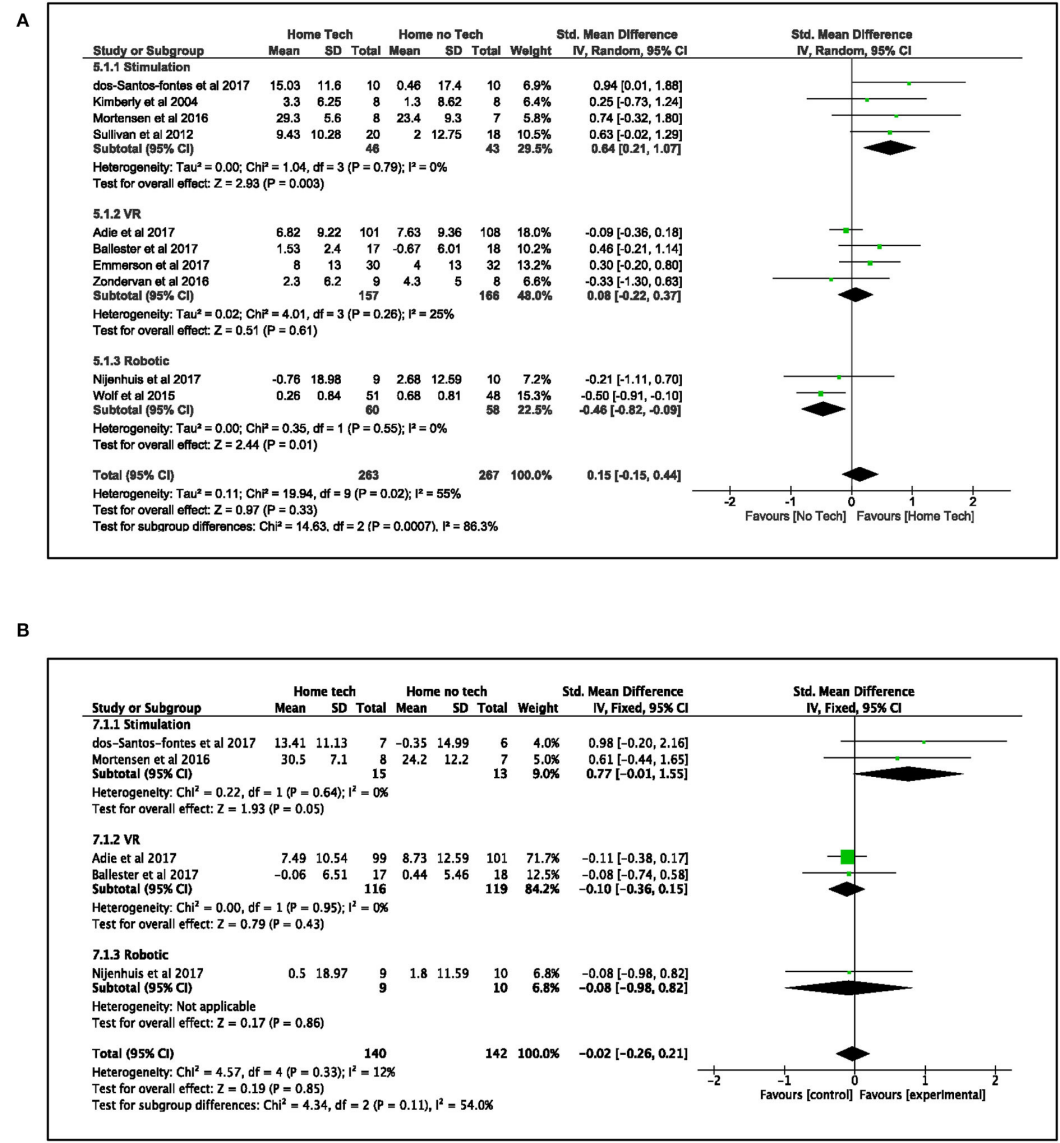
Comparison of the effect of technology-assisted home-based intervention and “no technology” intervention on UL function **(A)** standardized mean difference (SMD) immediately after treatment. **(B)** Standardized mean difference (SMD) at follow-up.

A pooled analysis of 10 studies (34, 35, 39, 40, 42, 43, 45, 50, 53, 55) that compared the effects of technology-assisted home-based interventions on upper limb function to “no technology” home-based interventions was conducted (see Figure 4). These studies used three broad categories of technology-assisted interventions: electrical stimulation (including transcranial direct stimulation), virtual reality, and robotics in the experimental groups. All the home-based interventions used in these studies except for one (43) are either self-directed or remotely supervised by a therapist. The overall effects showed similar improvements in both the technology-assisted home-based intervention groups and their control groups that used “no technology” intervention after treatment [SMD: 0.15, 95% CI (−0.15, 0.44), *I*^2^ = 55%, *p* = 0.33, random effect model] and at follow-up [SMD: −0.02, 95% CI (−0.26, 0.21), *I*^2^ = 12%, *p* = 0.85, fixed effect model]. The funnel plots (see Figures S4A,B in Supplementary Information 3) for both analyses were symmetrical with no publication bias supported by Egger's test (after treatment: β: 1.62, SE: 0.942, *p* = 0.125; follow-up: β: 1.35, SE: 0.67, *p* = 0.136).

Nevertheless, further subgroup analysis revealed differing results in the three categories of interventions. Interventions that used electrical stimulation (39, 42, 43, 50) demonstrated statistically significant benefits in improving the paretic upper limb function as compared to sham or task-specific training after treatment [SMD: 0.64, 95% CI (0.21, 1.07), *I*^2^ = 0%, *p* = 0.003, random effect model] and at follow-up [SMD: 0.77, 95% CI (−0.01, 1.55), *I*^2^ = 0%, *p* = 0.05, fixed effect model]. On the contrary, results from robotic studies (45, 53) favored their control groups that used the “no technology” home exercises program [SMD: −0.46, 95% CI (−0.82, −0.09), *I*^2^ = 0%, *p* = 0.01, random effect model] after treatment but not at follow-up [SMD: −0.08, 95% CI (−0.98, 0.82), *p* = 0.86, fixed effect model]. Results from virtual reality studies (34, 35, 40, 55) found no group differences between the virtual reality and control groups at the two time points: after treatment [VR: SMD: 0.08, 95% CI (−0.22, 0.37), *I*^2^ = 25%, *p* = 0.61, random effect model] and at follow-up [VR: SMD:−0.10, 95% CI (−0.36, 0.15), *I*^2^ = 0%, *p* = 0.43, fixed effect model].

**Figure 5:**
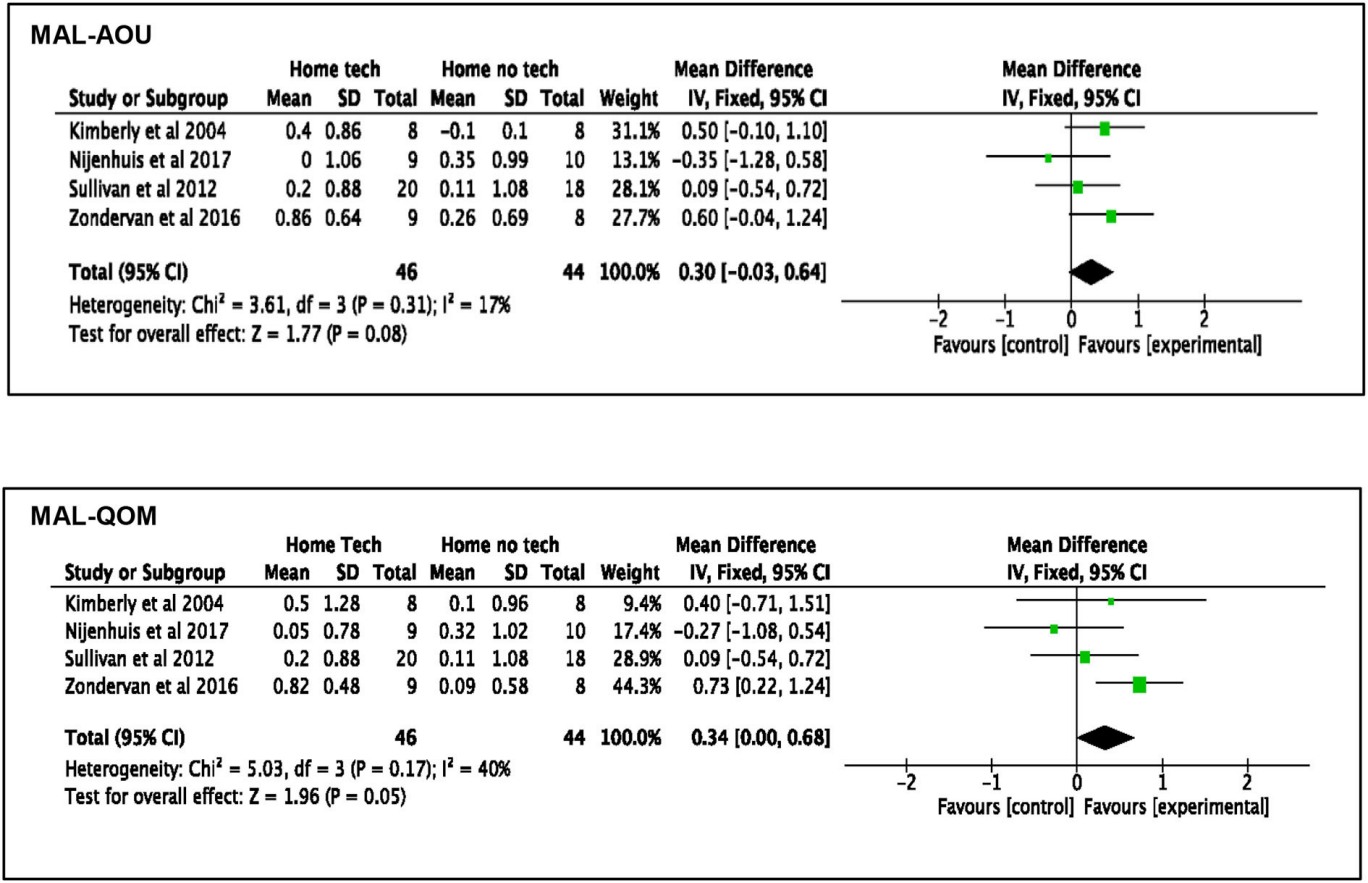
Comparison of the effect of the technology-assisted home-based intervention and “no technology” intervention on MAL outcomes immediately after treatment MAL-AOU and MAL-QOM.

Regarding the participants' perceived use of their paretic arm in daily activities, the meta-analysis of four studies (42, 45, 50, 55) found different pooled outcomes on the two subscales in MAL when examining the effect of technology-assisted interventions (see Figure 5). The technology-assisted interventions had a beneficial effect on the quality of arm movement (MAL-QOM) when compared to “no technology” interventions [MD: 0.34, 95% CI (0, 0.68), *I*^2^ = 40%, *p* = 0.05, fixed effect model]. However, the analysis narrowly failed to show a statistically significant benefit on the amount of use (MAL-AOU) [MD: 0.30, 95% CI (−0.03, 0.64), *I*^2^ = 17%, *p* = 0.08, fixed effect model]. Further analysis found that the work by Zondervan et al. (55) was the main contributor to the significant result in MAL-QOM. It implied that the observed pooled outcome of technology-assisted home intervention might not be conclusive.

#### Home-based UL intervention vs. no intervention

**Figure 6:**
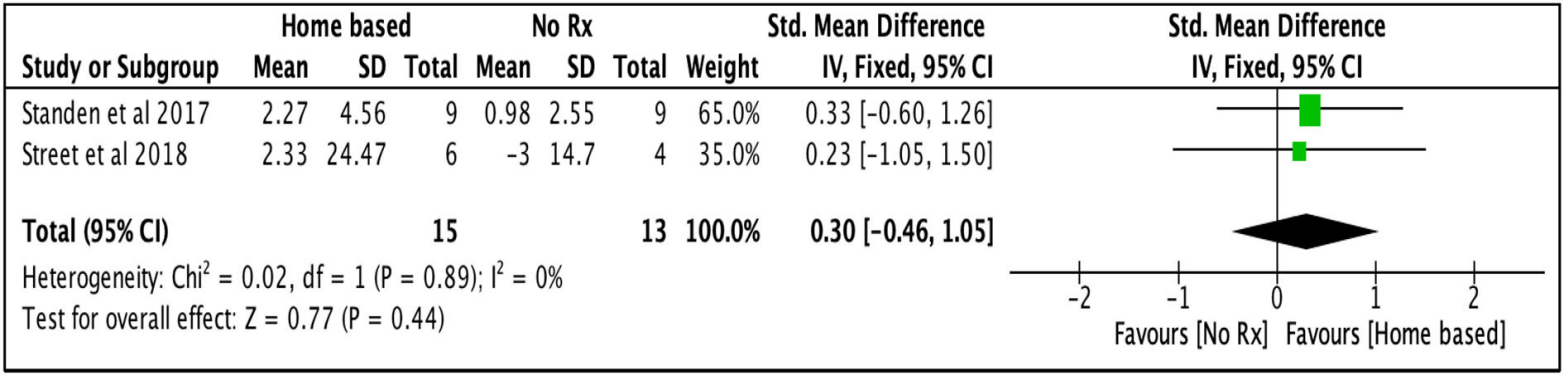
Comparison of the effect of home-based intervention and no treatment on UL function after the treatment.

Two studies (47, 48) had used no interventions in their control group compared to their technology-assisted home-based upper limb intervention. The pooled effect did not demonstrate a statistical significant benefit of home-based treatment over no treatment to improve UL function [SMD: 0.30, 95% CI (−0.46, 1.05), *I*^2^ = 0%, *p* = 0.44, fixed effect model; Figure 6]. A possible reason might be these studies' small effects and sample sizes.

## Discussion

This review summarizes the methodological qualities, content, and clinical effects of home-based upper limb rehabilitation in stroke rehabilitation. The characteristic of this review is that all the included studies were randomized controlled trials (RCT) and randomized cross-over trials, and the majority were high-quality trials. With clear clinical relevance and focus, the meta-analysis added rigor to the synthesis in evaluating the effectiveness of home-based interventions to improve upper limb function after stroke.

This review examined the effects of home-based upper limb rehabilitation on hemiparetic upper limb motor recovery in stroke survivors. Two key findings were highlighted in the meta-analysis: (1) home-based upper limb interventions were more effective in improving hemiparetic upper limb function and increasing participants' satisfaction in the use of the affected arm in daily activities than clinic-based therapy after treatment and at follow-up; (2) among the home-based interventions, those that used electrical stimulation were more effective in improving the hemiplegic arm's function than “no technology” intervention after treatment and follow-up.

The pooled evidence demonstrated that the home-based upper limb intervention was superior to clinic-based therapy in improving the hemiplegic arm's function after stroke. This finding is considered new compared to a previous Cochrane review (20). Due to the paucity of available studies, the review (20) found insufficient evidence (i.e., four RCTs) to determine the effects of home-based therapy programs for upper limb recovery in stroke survivors. In this review, the upper limb functional gains in participants who received the home-based intervention were consistent with their satisfaction with the affected arm's daily use, as reflected in their Motor Activity Log scores. This consistency between the participant's motor improvement and satisfaction illustrated the benefits of interventions conducted in the home setting. Previous evidence suggested a possible influence of the home environment on the treatment outcome (52, 58). Home-based rehabilitation provides contextual dependent learning and uses daily objects relevant to the patients (59, 60). Patients practiced in a familiar environment were likely to transfer skills learned in real-world activities (58). The clinic where conventional therapy was carried out served as a poor surrogate environment separating the person from the natural context (15). Transfer of skills and treatment effects from such an environment to a real-life situation might be inadequate and not feasible (15, 51). Waddell et al. (56) found that upper limb training designed to improve upper limb capacity in the clinic setting was insufficient to translate into actual improvements in upper limb performance in daily activities. One possible reason was that therapists who provide training in the clinic might be unaware of the constraints (or supports) in the person's real-life environment to assist the patients in translating skills (15). Therefore, it is recommended that therapists providing training to the patients outside of the home context should consider the patient's home environment setup.

Despite the overall positive effects of home-based upper limb intervention over conventional therapy, only the effect of “no technology” interventions was superior to conventional therapy in the clinic when a subgroup analysis was performed. The type of “no technology” interventions used is task-specific training, therapeutic exercises, and home-based CIMT. All these studies (17, 18, 36, 51, 52) involved direct contact with the therapist in their interventions. One possible explanation for the positive effect was that the studies (17, 18, 36, 51, 52) had used customized and relevant functional activities that matched the participants' goals and arm capacity. This method and direct contact with the therapist kept the participants engaged and compliant with the therapy regime, triggering positive results. In an unstructured setting like the home, interventions need relevance and command enough interest to keep patients motivated and engaged (21).

On the contrary, our review indicated that the effects of the home-based technology-assisted interventions were similar to clinic-based therapy. To highlight, all these interventions in these studies (8, 37, 38) were self-directed or remotely supervised by the therapist. One purpose of using home-based technologies was to reduce the need for direct contact with a therapist, ameliorating the saturated health services (61). Nonetheless, this observation from the subgroup analysis highlighted a critical consideration when choosing the type of technology-assisted home-based upper limb interventions. The use of home-based technologies requires additional consideration of a broader range of factors such as the individual's motivation, social context, technical proficiency, physical space, and the usability and therapeutic design of the technology devices (10, 34). Inadequate considerations of these factors might affect the patient's motivation and adherence to the therapy regime, especially in the absence of the therapist, thereby affecting the treatment outcomes (10).

Another key finding was that differential effects were found in different interventions after treatment and follow-up when comparing technology-assisted home-based intervention to “no technology” home-based intervention in the 10 included studies (34, 35, 39, 40, 42, 43, 45, 50, 53, 55). Unlike the “no technology” home-based interventions mentioned above, all the interventions (technology-assisted and “no technology”) in these studies were self-directed or remote supervised. Only one transcranial direct stimulation study (43) involved direct supervision by the therapist. Home-based electrical stimulation interventions were more effective than a sham or “no technology” intervention in improving UL function after treatment and follow-up. This result was consistent with previous reviews (21, 28). Anatomically, the upper limb has high tactile sensitivity, occupying a large area of the somatosensory homunculus (62). One proposed explanation for the positive effects of electrical stimulation was that it provides enhanced somatosensory input and increased cognitive sensory attention, which proved to be effective in improving the upper limb's performance in patients with stroke (39, 41). Previous studies highlighted a close relationship between the increased ipsilesional somatosensory cortex (S1) activation and motor improvements induced by training such as CIMT and electrical stimulation (42, 63). Most participants in this review were in the chronic stage of stroke (a mean stroke onset time of 23.5 ± 21.2 months). Previous studies (63, 64) had suggested that combining electrical stimulation with other interventions such as CIMT in patients with chronic stroke would enhance S1 excitability further. Future studies can consider exploring the effectiveness of such a combination conducted in the home to promote cortical reorganization. Another explanation is that the usability of the electrical stimulators used in this review significantly contributes to the positive effects. The electrical stimulators were portable and easy to use. These features allow the participant to manage the devices easily in the home setting (41) with minimal supervision.

Moreover, the effect of virtual reality was equivalent to “no technology” intervention in improving upper limb function in the home. This observation contradicts recent evidence that favored the effectiveness of virtual reality in improving arm function after stroke (65, 66). Virtual reality (VR) involves the interactive simulation of an environment, scenario, or activity created by a computer, allowing the user to interact through multiple sensory canals (67, 68). The Cochrane review (65) found that virtual reality was more beneficial when conducted in the first 6 months and used a minimal training dose of more than 15 hours. One difference was that most of the VR interventions in the Cochrane review (65) were conducted in a clinic and under therapists' supervision. Therapists used standardized approaches to guide the patients through therapy and motivate them to engage in treatment (10). Unlike all the virtual reality interventions in this review, which were mainly self-directed and conducted at home. The lack of a structured session and absence of therapists might reduce patients' engagement (10) and affect the treatment outcome.

The robotic studies showed contrasting outcomes among the other two mentioned interventions. The pooled results favored traditional home-based exercises without technology on the effect on upper limb function. Consistent with previous studies (6, 69, 70), robotic-assisted therapy's clinical effect was modest compared to conventional treatment. The robotic-assisted therapy used power-assisted robotic devices to allow fine graded upper limb movements and precise measurements (13). One explanation for this unfavorable result was the intensity of the treatment dose. Robotic technology is designed to provide intense, highly repetitive, and task-specific training (71). However, it was not reflected in Nijenhuis's study (45). Nijenhuis et al. (45) found that their control group had a higher training duration than the robotic group. The marked difference in training duration was attributed to the limited variety of exercises available in the robotic group (i.e., three exercises) vs. 34 exercises for the control group (45). Adherence to training duration is essential and more attention to motivational strategies is needed when using technology-assisted training (45). A large variety of attractive and functional exercises are crucial to prevent boredom and abandonment and increase adherence (7). Creating various customized exercises while keeping robotic devices affordable can be a potential challenge for robotic therapy. Nevertheless, this observation in the robotic studies necessitates caution in interpretation as there are only two robotic studies available for analysis, limiting the results' generalizability.

## Limitations and recommendations

Though the studies included in this review demonstrated a low risk of bias in terms of methodological quality, there was substantial heterogeneity between the studies clinically and statistically for one meta-analysis. The included studies varied in the types of interventions used and time of post-stroke onset among the participants. A range of upper limb outcome measures was used across the studies, making it difficult to compare. This review has included studies from January 2000 to September 2020. Trials before January 2000 and after September 2020 have not been reviewed.

The review's primary outcome focuses on upper limb motor function and use; other domains such as cost-effectiveness and compliance to interventions are not evaluated. Further studies are recommended to capture such domains. This review was unable to compare the use of remote-supervised therapy and self-directed therapy due to the limited number of studies using remote supervision (*n* = 4) and they varied in comparison and interventions used.

The use of the affected upper limb in daily activities was self-reported from the Motor Activity Log. Self-report measures are subject to many report biases, such as social desirability (72) and cognitive deficits (i.e., reliance on an individual's recall) (73). Further studies can consider using technology such as an accelerometer to capture arm use in daily activities objectively.

This review demonstrated that home-based UL interventions with direct supervision from the therapist were more effective than in-clinic therapy or technology-assisted interventions delivered in self-directed or remote supervision. Nevertheless, maintaining such a mode of therapy is not sustainable due to the increasing demand for rehabilitation services. Future studies can consider exploring the clinical and cost-effectiveness of a hybrid therapy model in which directly supervised therapy is kept to the minimum and supported with home-based technologies to carry the therapy in a self-directed or remotely supervised manner. Lastly, given the positive effects of home-based electrical stimulation, further studies can consider combining this intervention with home-based CIMT as proposed by previous studies (63, 64).

## Conclusion

The beneficial effects of home-based upper limb interventions were superior to conventional therapy in improving function and perceived use of the affected upper limb in daily activities. Nevertheless, in an unstructured environment like the home setting, the choice of home-based technology-assisted interventions requires careful consideration of the individual's physical environment, social context, technical proficiency, and motivation (10, 34). Among the home-based interventions, home-based electrical stimulation seemed to provide the most optimal benefits compared to conventional treatment in the home setting.

## Clinical messages

◦ Home-based upper limb interventions are more effective than conventional therapy to improve the arm function as it provides contextual learning for better translation of skills to the real-life domain.
◦ When selecting the types of technology-assisted interventions in the home setting, careful considerations on factors such as one's motivation, social context, technical proficiency, physical environment and the therapeutic design and usability of the devices are required.
◦ The somatosensory input from the electrical stimulation seems to provide the optimal benefits among the home-based upper limb interventions.

## Data availability statement

The datasets presented in this article are not readily available because this is a systematic review and meta-analysis, and does not contain any raw data. Requests to access the datasets should be directed to rsnkfong@polyu.edu.hk.

## Author contributions

SFMT and KNKF: study objective. SFMT and PFC: literature search and data extraction. SFMT, KNKF, and PFC: methodological quality assessment, critical review, and approval of the manuscript. All authors have read and approved the final manuscript.

## Funding

This research project is funded by the Research Impact Fund (Ref. no.: R5028-20F) to KNKF, Research Grants Council, University Grants Committee, Hong Kong SAR, China.

## Conflict of interest

The authors declare that the research was conducted in the absence of any commercial or financial relationships that could be construed as a potential conflict of interest.

## Publisher's note

All claims expressed in this article are solely those of the authors and do not necessarily represent those of their affiliated organizations, or those of the publisher, the editors and the reviewers. Any product that may be evaluated in this article, or claim that may be made by its manufacturer, is not guaranteed or endorsed by the publisher.

## Abbreviations

CINAHL: Cumulated Index to Nursing and Allied Health Literature
SMD: standardized mean difference
MD: mean difference
UL: upper limb
CIMT: constraint-induced movement therapy
ADL: activities of daily living
RCT: randomized controlled trial
Pedro: Physiotherapy Evidence Database
SD: standard deviation
IQR: interquartile ratio
MT: mirror therapy
FMA-UE: Fugl-Meyer Assessment-Upper Extremity
ROM: range of motion
ARAT: Action Research Arm Test
WMFT: Wolf Motor Function Test
BBT: Box and Block Test
9-HPT: Nine-hole Peg Test
JTT: Jebsen–Taylor test
MAL-AOU: Motor Activity Log-amount of use
MAL-QOM: Motor Activity Log-quality of movement
CI: confidence interval
VR: virtual reality.
