# Supplementary material for: Effectiveness of home-based upper limb rehabilitation in stroke survivors: A systematic review and meta-analysis
Source: Front Neurol. 2022 Sep 9;13:964196. doi: 10.3389/fneur.2022.964196 (PMC9521568; doi:10.3389/fneur.2022.964196)
Supplement: Supplementary file 1 [file Data_Sheet_1.PDF]

Supplementary information 1

Review question

1. To determine whether home-based upper limb rehabilitation intervention is effective in improving upper limb recovery of stroke survivors as compared to conventional therapy or placebo or no treatment.
2. To identify the types of home-based upper limb intervention with optimal benefits to improve upper limb (UL) function after stroke.

**Formulating questions using PICO**			
**Population (P)**	**Intervention (I)**	**Comparison (C)**	**Outcome (O)**
Stroke patients	Home-based upper limb intervention	❖ Conventional therapy, or placebo<br><br>Define as<br>• Placebo or no treatment<br>• Conventional therapy is considered as normal or usual component of stroke rehabilitation, i.e. Centre/clinic-based or outpatient training or Inpatient training<br>• Intervention delivered in hospital or clinic setting<br>• Including alternative treatment (e.g. acupuncture)<br><br>❖ Another form of home-based UL intervention	Upper limb motor and functional outcomes

Supplementary information 1

Keywords

No	Keywords	Variation	Combination
1.	Home-based	a. Home-based	(1a or 1b or 1c or 1d) AND (2a or 2b or 2c or 2d or 2e or 2f or 2g) AND (3a or 3b or 3c) AND (4a or 4b or 4c or 4d)
		b. Home$/*	
		c. House$/*	
		d. Residential$/*	
2.	Upper limb	a. Upper limb*/$	
		b. Upper extremity*/$	
		c. Arm$/*	
		d. Hemiplegia$/*	
		e. Hand$/*	
		f. Paretic$/*	
		g. Paresis $/*	
3.	Rehabilitation	a. Rehabilitation*/$	
		b. Therapy$/*	
		c. Intervention$/*	
		d. Physiotherapy$	
4.	Stroke	a. Stroke */$	
		b. Cerebrovascular disease	
		c. Cerebral vascular accident */$	
		d. Transient ischaemic attack */$	

Supplementary information 1

DATABASE: CINAL (54)

Search ID#	Search Terms	Search Options	Actions
S7	S4 AND S6	**Expanders** - Apply equivalent subjects<br>**Search modes** - Boolean/Phrase	**View Results** (54)
S6	S3 AND S5	**Expanders** - Apply equivalent subjects<br>**Search modes** - Boolean/Phrase	**View Results** (332)
S5	S1 AND S2	**Expanders** - Apply equivalent subjects<br>**Search modes** - Boolean/Phrase	**View Results** (510)
S4	(stroke$ or cerebrovascular accident or cva) OR cerebral vascular accident OR transient ischemic attack	**Limiters** - Full Text; Published Date: 20000101-20200931; English Language; Human; Age Groups: All Adult<br>**Expanders** - Apply equivalent subjects<br>**Search modes** - Boolean/Phrase	**View Results** (5,553)
S3	(rehabilitation or therapy or treatment or intervention) OR (physical therapy or physiotherapy) OR interventions$	**Limiters** - Full Text; Published Date: 20000101-20200931; English Language; Human; Age Groups: All Adult<br>**Expanders** - Apply equivalent subjects<br>**Search modes** - Boolean/Phrase	**View Results** (99,961)
S2	(upper limb$ or upper extremity$ or arm or hand) OR (hemiplegia or hemiparesis or hemiparetic or hemiplegic) OR (paresis or paretic)	**Limiters** - Full Text; Published Date: 20000101-20200931; English Language; Human; Age Groups: All Adult<br>**Expanders** - Apply equivalent subjects<br>**Search modes** - Boolean/Phrase	**View Results** (8,174)
S1	(home based$ or home$ or house$) OR residential$	**Limiters** - Full Text; Published Date: 20000101-20200931; English Language; Human; Age Groups: All Adult<br>**Expanders** - Apply equivalent subjects	**View Results** (12,545)

Supplementary information 1

DATABASE: Medline (84)

Select / deselect all | Search with AND | Search with OR | Delete Searches | Refresh Search Results

Search ID#	Search Terms	Search Options	Actions
S7	S4 AND S6	**Expanders** - Apply equivalent subjects<br>**Search modes** - Boolean/Phrase	View Results (84) | View Details | Edit
S6	S3 AND S5	**Expanders** - Apply equivalent subjects<br>**Search modes** - Boolean/Phrase	View Results (655) | View Details | Edit
S5	S1 AND S2	**Expanders** - Apply equivalent subjects<br>**Search modes** - Boolean/Phrase	View Results (1,004) | View Details | Edit
S4	(stroke or cerebrovascular accident or cva) OR transient ischemic attack OR cerebral vascular accident	**Limiters** - Full Text; Date of Publication: 20000101-20200931; Human; Age Related: All Adult: 19+ years<br>**Expanders** - Apply equivalent subjects<br>**Search modes** - Boolean/Phrase	View Results (15,774) | View Details | Edit
S3	(rehabilitation or therapy) OR intervention OR treatment OR physiotherapy or physical therapy	**Limiters** - Full Text; Date of Publication: 20000101-20200931; Human; Age Related: All Adult: 19+ years<br>**Expanders** - Apply equivalent subjects<br>**Search modes** - Boolean/Phrase	View Results (309,233) | View Details | Edit
S2	upper limb$ OR (upper extremity or hand or arm) OR (hemiplegia$ or hemiparesis) OR (paretic or paresis)	**Limiters** - Full Text; Date of Publication: 20000101-20200931; Human; Age Related: All Adult: 19+ years<br>**Expanders** - Apply equivalent subjects<br>**Search modes** - Boolean/Phrase	View Results (27,385) | View Details | Edit
S1	home$ OR house$ OR residential$ or home based	**Limiters** - Full Text; Date of Publication: 20000101-20200931; Human; Age Related: All Adult: 19+ years<br>**Expanders** - Apply equivalent subjects<br>**Search modes** - Boolean/Phrase	View Results (22,218) | View Details | Edit

Supplementary information 1

DATABASE: Web of Science (899)

Set	Results	Save History / Create Alert · Open Saved History	Edit Sets	Combine Sets AND OR Combine	Delete Sets Select All ✕ Delete
# 10	899	(#9 AND #8 AND #7 AND #6) *AND* **LANGUAGE:** (English) *AND* **DOCUMENT TYPES:** (Article)<br>*Indexes=SCI-EXPANDED, SSCI, A&HCI, CPCI-S, CPCI-SSH, ESCI Timespan=2000-2020*	Edit	☐	☐
# 9	1,479,192	**TOPIC:** (upper limb* or upper extremity* or arm* or hand*) *OR* **TOPIC:** (hemiparesis* or hemiplegia* or hemiparetic* or hemiparesis* or hemiplegic*) *OR* **TOPIC:** (paretic* or paresis*)<br>*Indexes=SCI-EXPANDED, SSCI, A&HCI, CPCI-S, CPCI-SSH, ESCI Timespan=2000-2020*	Edit	☐	☐
# 8	5,765,465	**TOPIC:** (rehabilitation* or intervention* or treatment* or therapy*) *OR* **TOPIC:** (physiotherapy*) *OR* **TOPIC:** (physical therapy *)<br>*Indexes=SCI-EXPANDED, SSCI, A&HCI, CPCI-S, CPCI-SSH, ESCI Timespan=2000-2020*	Edit	☐	☐
# 7	996,041	**TOPIC:** (home-based* or home* or house*) *OR* **TOPIC:** (residential*)<br>*Indexes=SCI-EXPANDED, SSCI, A&HCI, CPCI-S, CPCI-SSH, ESCI Timespan=2000-2020*	Edit	☐	☐
# 6	332,351	**TOPIC:** (stroke* or cerebrovascular disease* or cva) *OR* **TOPIC:** (cerebral vascular accident*) *OR* **TOPIC:** (transient ischemic attack*)<br>*Indexes=SCI-EXPANDED, SSCI, A&HCI, CPCI-S, CPCI-SSH, ESCI Timespan=2000-2020*	Edit	☐	☐

Supplementary information 1

DATABASE: Cochrane (11)

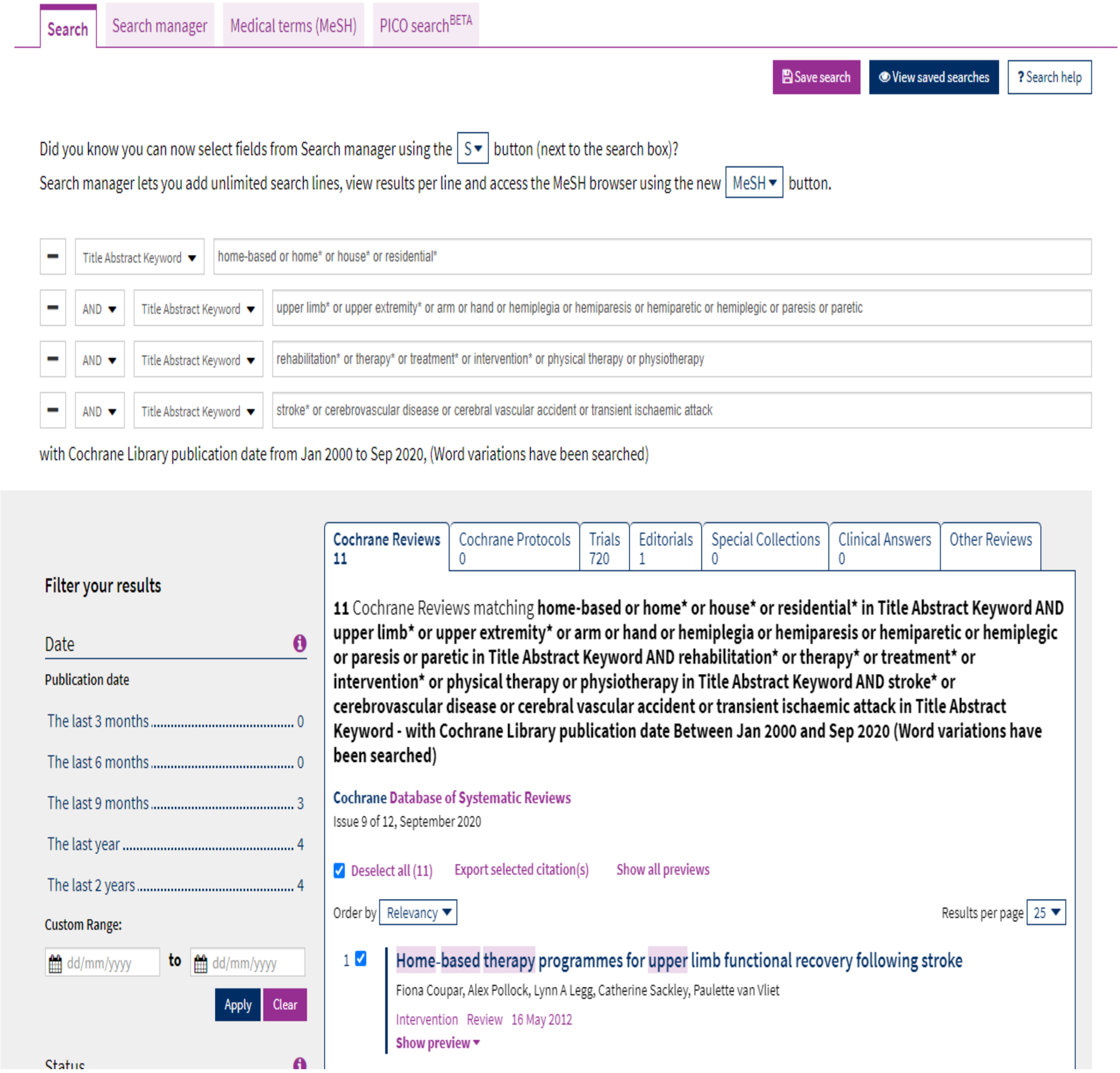

Search | Search manager | Medical terms (MeSH) | PICO search BETA

Save search | View saved searches | ? Search help

Did you know you can now select fields from Search manager using the S button (next to the search box)?
Search manager lets you add unlimited search lines, view results per line and access the MeSH browser using the new MeSH button.

Title Abstract Keyword: home-based or home* or house* or residential*
AND Title Abstract Keyword: upper limb* or upper extremity* or arm or hand or hemiplegia or hemiparesis or hemiparetic or hemiplegic or paresis or paretic
AND Title Abstract Keyword: rehabilitation* or therapy* or treatment* or intervention* or physical therapy or physiotherapy
AND Title Abstract Keyword: stroke* or cerebrovascular disease or cerebral vascular accident or transient ischaemic attack

with Cochrane Library publication date from Jan 2000 to Sep 2020, (Word variations have been searched)

Cochrane Reviews 11 | Cochrane Protocols 0 | Trials 720 | Editorials 1 | Special Collections 0 | Clinical Answers 0 | Other Reviews

Filter your results

Date

Publication date

The last 3 months ... 0
The last 6 months ... 0
The last 9 months ... 3
The last year ... 4
The last 2 years ... 4

Custom Range:

dd/mm/yyyy to dd/mm/yyyy

Apply Clear

Status

11 Cochrane Reviews matching **home-based or home* or house* or residential* in Title Abstract Keyword AND upper limb* or upper extremity* or arm or hand or hemiplegia or hemiparesis or hemiparetic or hemiplegic or paresis or paretic in Title Abstract Keyword AND rehabilitation* or therapy* or treatment* or intervention* or physical therapy or physiotherapy in Title Abstract Keyword AND stroke* or cerebrovascular disease or cerebral vascular accident or transient ischaemic attack in Title Abstract Keyword - with Cochrane Library publication date Between Jan 2000 and Sep 2020 (Word variations have been searched)**

Cochrane Database of Systematic Reviews
Issue 9 of 12, September 2020

Deselect all (11) | Export selected citation(s) | Show all previews

Order by Relevancy | Results per page 25

1 Home-based therapy programmes for upper limb functional recovery following stroke
Fiona Coupar, Alex Pollock, Lynn A Legg, Catherine Sackley, Paulette van Vliet
Intervention Review 16 May 2012
Show preview
