# Supplementary material for: Effectiveness of home-based upper limb rehabilitation in stroke survivors: A systematic review and meta-analysis
Source: Front Neurol. 2022 Sep 9;13:964196. doi: 10.3389/fneur.2022.964196 (PMC9521568; doi:10.3389/fneur.2022.964196)
Supplement: Supplementary file 2 [file Data_Sheet_2.PDF]

Supplementary information 2

Characteristics of excluded studies

No	Study	Reason for exclusion
**1**	Alon et al. 2007	- Intervention not specific delivered at home
**2**	Amasyali et al. 2016	- Intervention not specific delivered at home
**3**	Benvenuti et al. 2014	- Intervention not specific delivered at home<br>- Telerehabilitation delivered in kiosk vs usual outpatient care unclear that 80% of intervention performed at home
**4**	Bordoloi et al. 2019	- Only evaluate components of same home-based intervention
**5**	Brunner et al. 2012	- Intervention not specific delivered at home
**6**	Chen et al. 2017	- Primary outcome measure is not on UL motor function
**7**	de Diego et al. 2013	- Intervention not specific delivered at home
**8**	Geller et al. 2016	- This article is a supplement without full text, and study was not completed
**9**	Graef et al. 2016	- Only evaluate components of same home-based intervention
**10**	Hayner et al. 2010	- Intervention not specific delivered at home
**11**	Harris et al. 2009	- Not home-based intervention, inpatient
**12**	Huijgen et al. 2008	- Participants not specific to stroke
**13**	In et al. 2012	- Intervention not specific delivered at home

Supplementary information 2

**14**	Khan, et al. 2019	- Not home-based intervention
**15**	Kimberley et al. 2018	- Not home-based intervention

Supplementary information 2

No	Study	Reason for exclusion
**16**	Knutson et al. 2012	- Not fully home-based intervention, mix of clinic rehab
**17**	Knutson et al. 2016	- Intervention not specific delivered at home<br>- Involved laboratory practice (>60%)
**18**	Knutson et al. 2020	- Intervention not specific delivered at home<br>- Involved laboratory practice (>60%)
**19**	Lemmens et al. 2014	- Intervention not specific delivered at home
**20**	Levy et al. 2019	- Sub study, no comparison group
**21**	Li et al. 2019	- Intervention not specific delivered at home<br>- Involved hospital based practice (60%)
**22**	Lima et al. 2014	- Only evaluate components of same home-based intervention
**23**	McNulty et al. 2015	- Intervention not specific delivered at home<br>- Involved research institute (60%)
**24**	Michaelsen et al. 2006	- Only evaluate components of same home-based intervention
**25**	Mugler 2019	- Intervention not specific delivered at home
**26**	Piron 2008	- No full text found
**27**	Ring 2005	- Intervention not specific delivered at home
**28**	Souza 2015	- Intervention not specific delivered at home<br>- 50% in outpatient facility in treatment group vs 100% outpatient CIMT

Supplementary information 2

**29**	Thielbar et al. 2020	- Only evaluate components of same home-based intervention
**30**	Wittmann et al. 2016	- Not RCT, no comparison group<br>- Pre post study

Supplementary information 2

References for excluded studies

1. Alon G, Levitt AF, McCarthy PA. Functional electrical stimulation enhancement of upper extremity functional recovery during stroke rehabilitation: a pilot study. NNR. 2007;21(3):207-15.

2. Amasyali SY, Yaliman A. Comparison of the effects of mirror therapy and electromyography-triggered neuromuscular stimulation on hand functions in stroke patients: a pilot study. Int J Rehabil Res. 2016;39(4):302-7.

3. Benvenuti F, Stuart M, Cappena V, Gabella S, Corsi S, Taviani A, et al. Community-based exercise for upper limb paresis: a controlled trial with telerehabilitation.NNR. 2014;28(7):611-20.

4. Bordoloi K, Deka RS. Effectiveness of home exercise program with modified ROOD'S approach on muscle strength in post cerebral haemorrhagic individuals of Assam. A randomised trial. Int. J. Physiother. 2019;6(5):231-9.

5. Brunner IC, Skouen JS, Strand LI. Is modified constraint-induced movement therapy more effective than bimanual training in improving arm motor function in the subacute phase post stroke? A randomized controlled trial. Clin. Rehabil. 2012;26(12):1078-86.

6. Chen J, Jin W, Dong WS, Jin Y, Qiao FL, Zhou YF, et al. Effects of home-based telesupervising rehabilitation on physical function for stroke survivors with hemiplegia: a randomized controlled trial. Am J Phys Med Rehabil. 2017;96(3):152-60.

7. de Diego C, Puig S, Navarro X. A sensorimotor stimulation program for rehabilitation of chronic stroke patients. Restor. Neurol. Neurosci. 2013;31(4):361-71.

8. Geller D, Nilsen D, Gillen G, Vanlew S, Bernardo M. A randomized controlled comparative effectiveness pilot study on unimanual and bimanual mirror therapy as a home program. Am. J. Occup.Ther. 2016;70:1.

9. Graef P, Michaelsen SM, Dadalt MLR, Rodrigues D, Pereira F, Pagnussat AS. Effects of functional and analytical strength training on upper-extremity activity after stroke: a randomized controlled trial. Braz. J. Phys. Ther. 2016;20(6):543-52.

10. Hayner K, Gibson G, Giles GM. Comparison of constraint-Induced movement therapy and bilateral treatment of equal intensity in people with chronic upper-extremity dysfunction after cerebrovascular accident. Am. J. Occup.Ther. 2010;64(4):528-39.

11. Harris JE, Eng JJ, Miller WC, Dawson AS. A self-administered Graded Repetitive Arm Supplementary Program (GRASP) improves arm function during inpatient stroke rehabilitation: a multi-site randomized controlled trial. Stroke. 2009;40(6):2123-8.

12. Huijgen BCH, Volienbroek-Hutten MMR, Zampolini M, Opisso E, Bernabeu M, Van Nieuwenhoven J, et al. Feasibility of a home-based telerehabilitation system compared to usual care: arm/hand function in patients with stroke, traumatic brain injury and multiple sclerosis. J Telemed Telecare. 2008;14(5):249-56.

13. In TS, Jung KS, Lee SW, Song CH. Virtual reality reflection therapy improves motor recovery and motor function in the upper extremities of people with chronic stroke. J. Phys. Ther. Sci. 2012;24(4):339-43.

14. Khan F, Rathore C, Kate M, Joy J, Zachariah G, Vincent PC, et al. The comparative efficacy of theta burst stimulation or functional electrical stimulation when combined with physical therapy after stroke: a randomized controlled trial. Clin. Rehabil. 2019;33(4):693-703.

15. Kimberley TJ, Pierce D, Prudente CN, Francisco GE, Yozbatiran N, Smith P, et al. Vagus nerve stimulation paired with upper limb rehabilitation after chronic stroke: a blinded randomized pilot study. Stroke. 2018;49(11):2789-92.

16. Knutson JS, Chae J, Hart RL, Keith MW, Hoyen HA, Harley MY, et al. Implanted neuroprosthesis for assisting arm and hand function after stroke: a case study. J Rehabil Res Dev. 2012;49(10):1505-16.

17. Knutson JS, Gunzler DD, Wilson RD, Chae J. Contralaterally controlled functional electrical stimulation improves hand dexterity in chronic hemiparesis: a randomized trial. Stroke. 2016;47(10):2596-602.

18. Knutson JS, Makowski NS, Harley MY, Hisel TZ, Gunzler DD, Wilson RD, et al. Adding contralaterally controlled electrical stimulation of the triceps to contralaterally controlled functional electrical stimulation of the

finger extensors reduces upper limb impairment and improves reachable workspace but not dexterity: a randomized controlled trial. Am J Phys Med Rehabil. 2020;99(6):514-21.

19. Lemmens RJM, Timmermans AAA, Janssen-Potten YJM, Pulles SANTD, Geers RPJ, Bakx WGM, et al. Accelerometry measuring the outcome of robot-supported upper limb training in chronic stroke: a randomized controlled trial. PLOS ONE. 2014;9(5):e96414.

20. Levy T, Killington M, Lannin N, Crotty M. Viability of using a computer tablet to monitor an upper limb home exercise program in stroke. Physiother. Theory Pract. 2019.

21. Li Y-c, Wu C-y, Hsieh Y-w, Lin K-c, Yao G, Chen C-l, et al. The priming effects of mirror visual feedback on bilateral task practice: a randomized controlled study. Occup. Ther. Int. 2019:1-9.

22. Lima RCM, Michaelsen SM, Nascimento LR, Polese JC, Pereira ND, Teixeira-Salmela LF. Addition of trunk restraint to home-based modified constraint-induced movement therapy does not bring additional benefits in chronic stroke individuals with mild and moderate upper limb impairments: a pilot randomized controlled trial. NeuroRehabilitation. 2014;35(3):391-404.

23. McNulty PA, Thompson-Butel AG, Faux SG, Lin G, Katrak PH, Harris LR, Shiner CT. The efficacy of Wii-based Movement Therapy for upper limb rehabilitation in the chronic poststroke period: a randomized controlled trial. Int J Stroke INT J STROKE. 2015;10(8):1253-60

24. Michaelsen SM, Dannenbaum R, Levin MF. Task-specific training with trunk restraint on arm recovery in stroke – randomized control trial. Stroke. 2006;37(1):186-92.

25. Mugler EM, Tomic G, Singh A, Hameed S, Lindberg EW, Gaide J, et al. Myoelectric computer interface training for reducing co-activation and enhancing arm movement in chronic stroke survivors: a randomized trial. NNR. 2019;33(4):284-95.

26. Piron L, Turolla A, Tonin P, Piccione F, Lain L, Dam M. Satisfaction with care in post-stroke patients undergoing a telerehabilitation programme at home. J Telemed Telecare. 2008;14(5):257-60.

27. Ring H, Rosenthal N. Controlled study of neuroprosthetic functional electrical stimulation in sub-acute post-stroke rehabilitation. J Rehabil Med. 2005;37(1):32-6.

28. Souza WC, Conforto AB, Orsini M, Stern A, Andre C. Similar effects of two modified constraint-induced therapy protocols on motor impairment, motor function and quality of life in patients with chronic stroke. Neurol. Int. 2015;7(1).

29. Thielbar KO, Triandafilou KM, Barry AJ, Yuan N, Nishimoto A, Johnson J, et al. Home-based upper extremity stroke therapy using a multiuser virtual reality environment: a randomized trial. Arch. Phys. Med. Rehabil. 2020;101(2):196-203.

30. Wittmann F, Held JP, Lambercy O, Starkey ML, Curt A, Hover R, et al. Self-directed arm therapy at home after stroke with a sensor-based virtual reality training system. J. Neuroeng. Rehabilitation. 2016;13.
