# Supplementary material for: Effectiveness of home-based upper limb rehabilitation in stroke survivors: A systematic review and meta-analysis
Source: Front Neurol. 2022 Sep 9;13:964196. doi: 10.3389/fneur.2022.964196 (PMC9521568; doi:10.3389/fneur.2022.964196)

Supplementary information 3

Funnel Plots for Meta-analysis results

Figure S2A: Funnel plot for comparison of home-based intervention and conventional therapy on UL function immediately after treatment

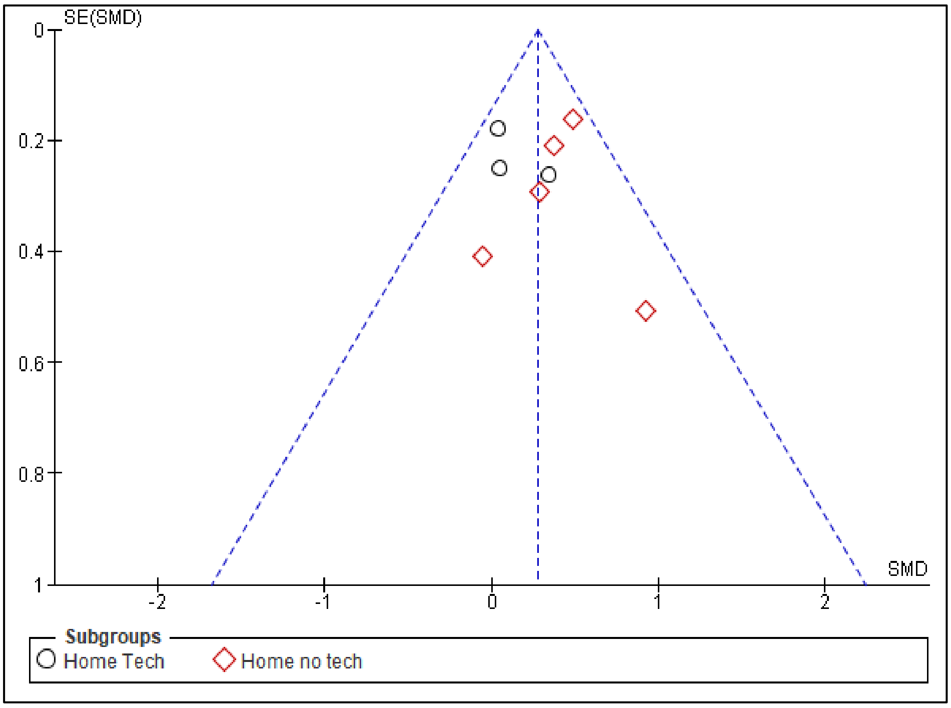

Egger's regression value (effect size): β: 0.04, standard error: 1.24, p=0.98

Figure S2B: Funnel plot for comparison of home-based intervention and conventional therapy on UL function at follow-up

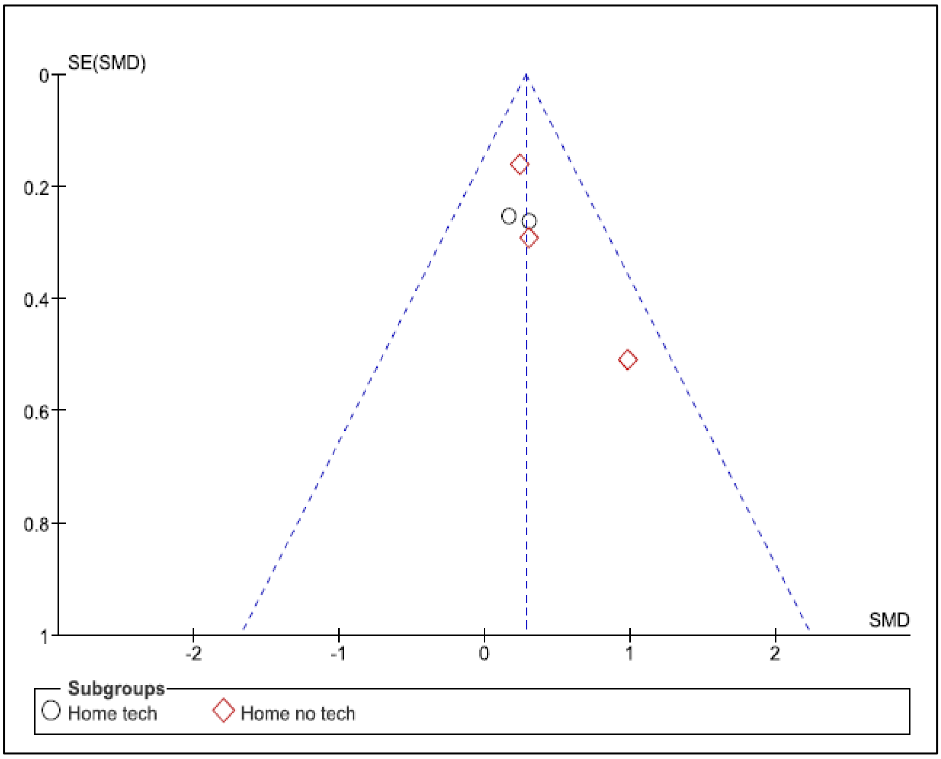

Egger's regression value (effect size) β: 1.56, standard error: 0.72, p=0.12

Supplementary information 3

Figure S3A: Funnel plot for comparison of home-based intervention and clinic-based intervention on self-reported amount of UL use (MAL-AOU) and quality of movement (QOM) immediately after treatment

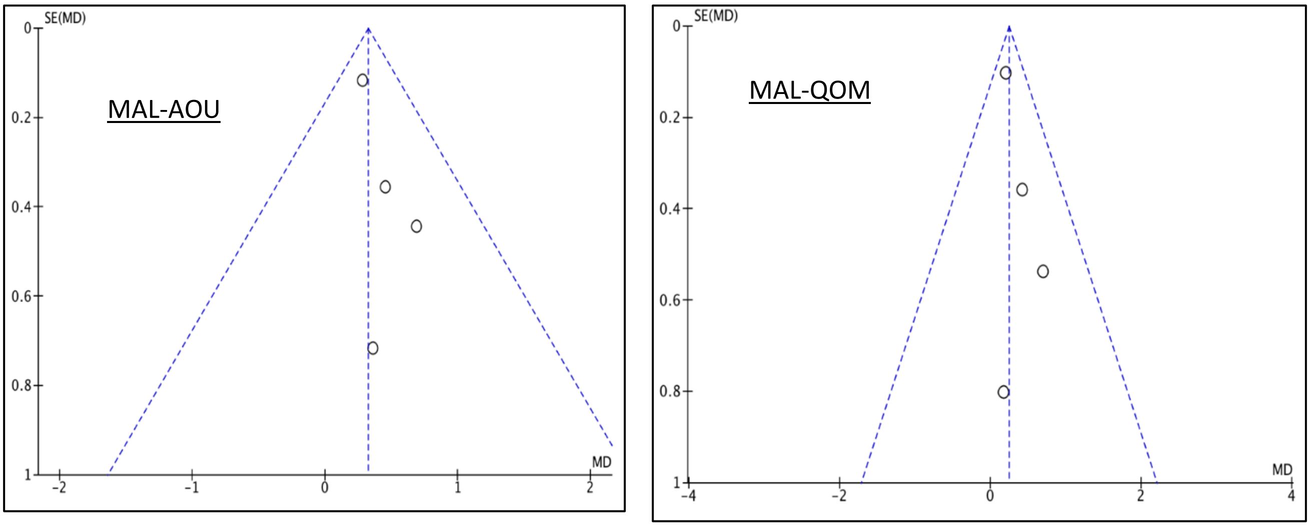

Figure S3B: Funnel plot for comparison of home-based intervention and conventional therapy on self-reported UL use MAL-AOU & MAL-QOM at follow-up

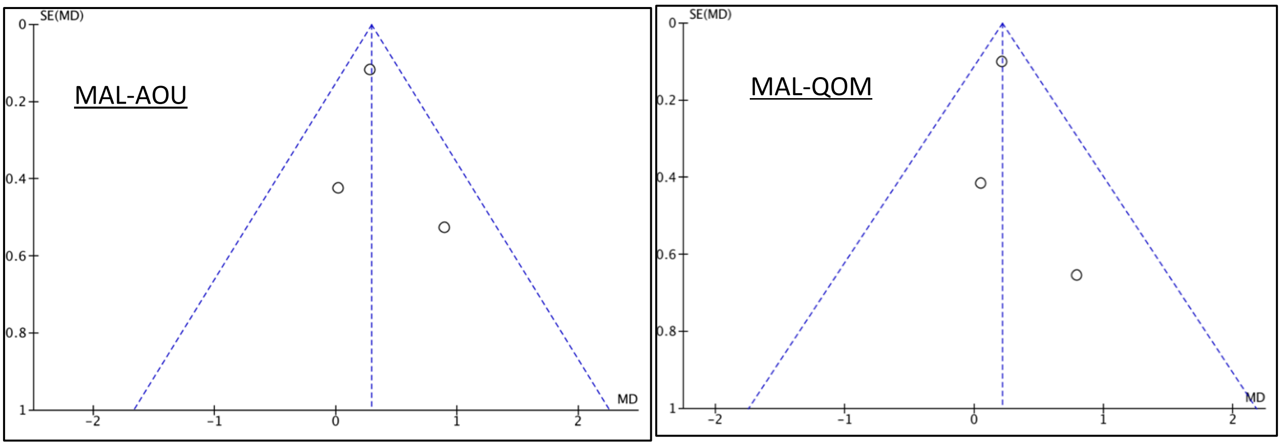

Supplementary information 3

Figure S4A: Funnel plot for comparison of technology-assisted home-based intervention and no technology home-intervention on UL function immediately after treatment

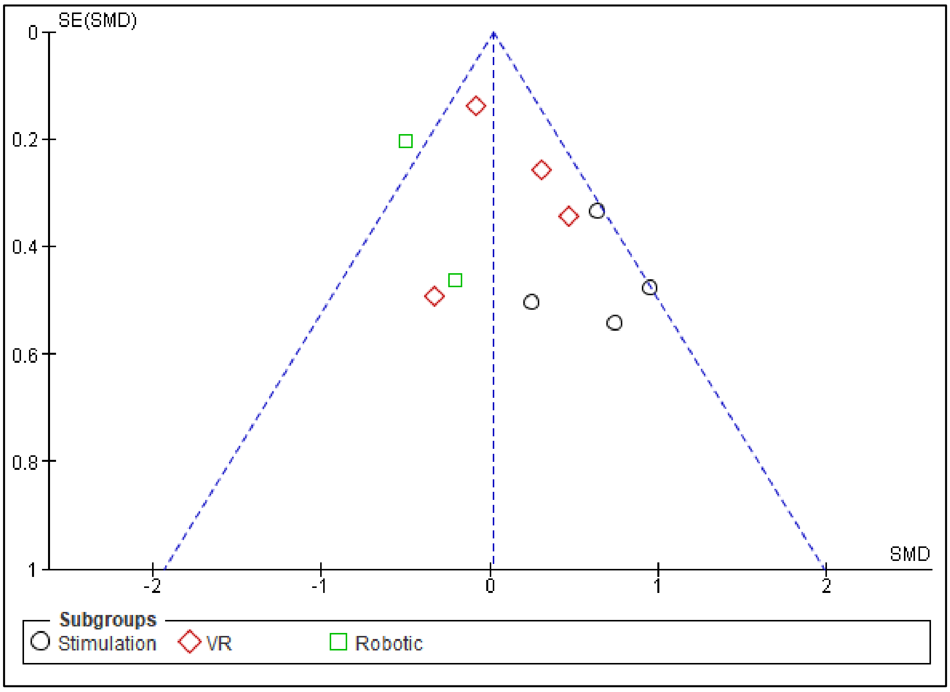

Egger's regression value (effect size): β: 1.62, standard error: 0.942, p=0.125

Figure S4B: Funnel plot for comparison of technology-assisted home-based intervention and no technology home-intervention on UL function at follow-up

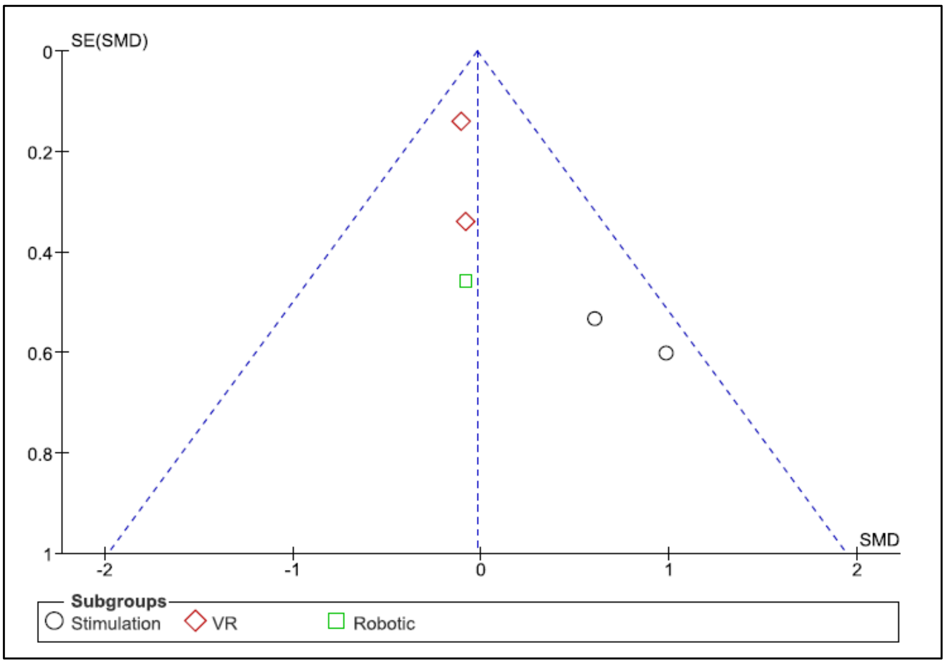

Egger's regression value (effect size): β: 1.35, standard error: 0.67, p=0.136

Supplementary information 3

Figure S5: Funnel plot for comparison of technology-assisted home-based intervention and no technology home-based intervention self-reported UL use: MAL-AOU & MAL-QOM immediately after treatment

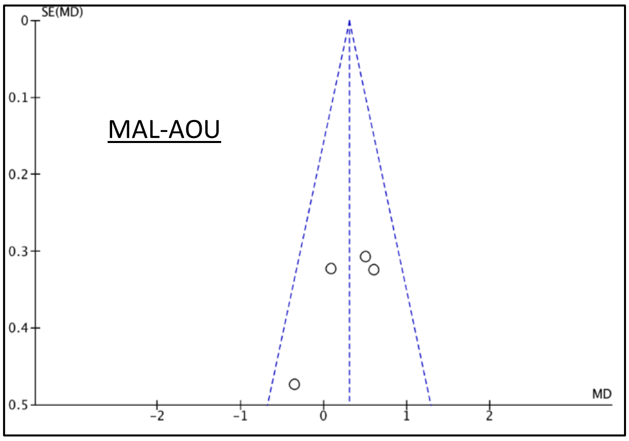

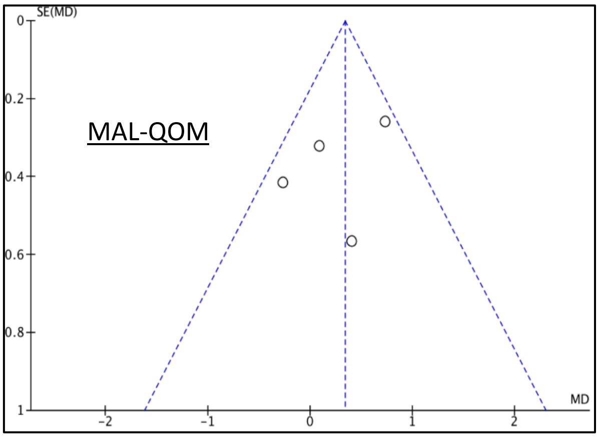

Figure S6: Comparison of home-based intervention and no intervention on UL function

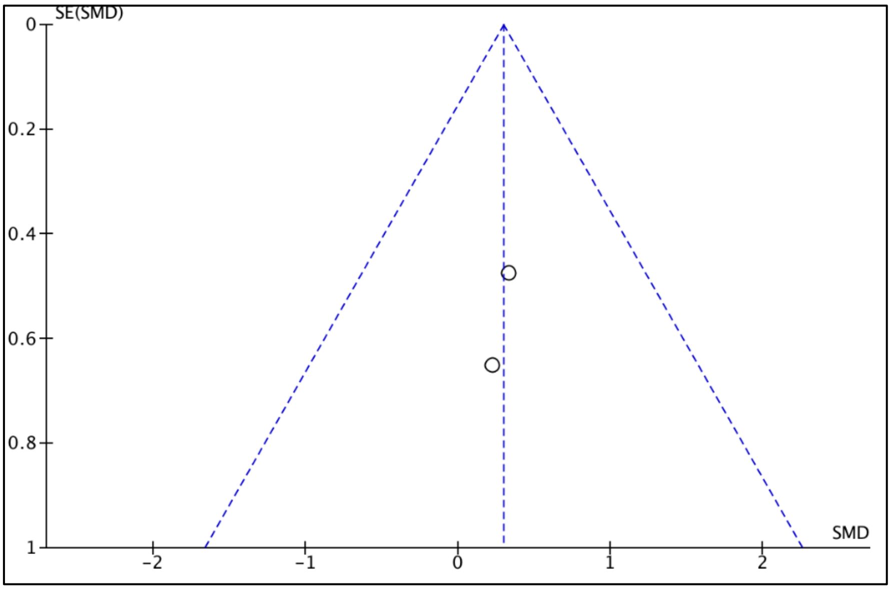

Supplement: Supplementary file 3 [file Data_Sheet_3.PDF]
